# Supplementary figures and images for: Contextual inference through flexible integration of environmental features and behavioural outcomes
Source: PLoS Comput Biol. 2026 Mar 20;22(3):e1014093. doi: 10.1371/journal.pcbi.1014093 (PMC13029755; doi:10.1371/journal.pcbi.1014093)

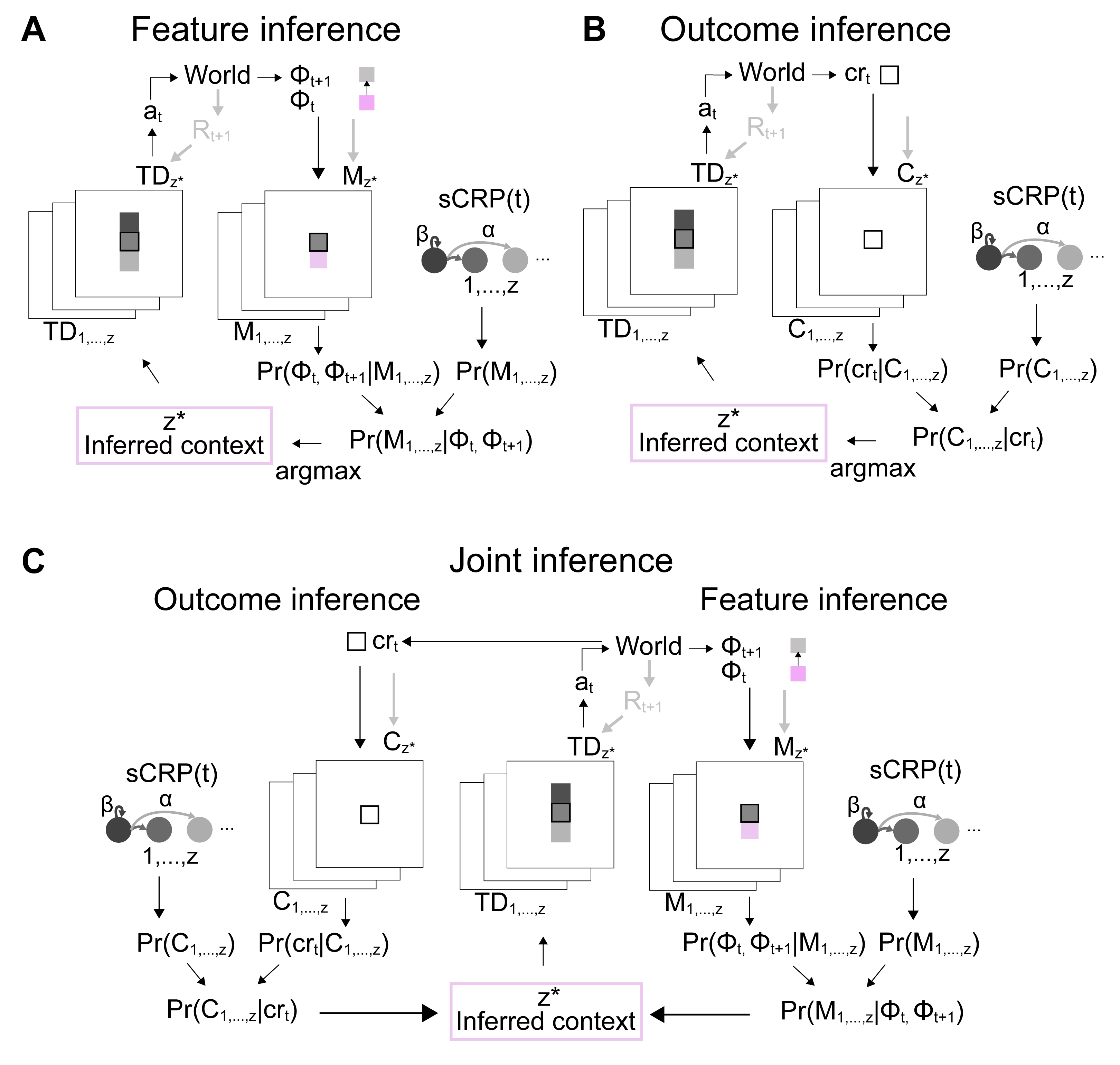

Supplement: S1 Fig — (A) Schematic of HPC model, showing how observed feature transitions are compared with existing successor feature maps using Bayesian inference to determine which context is currently most likely, (B) Schematic of PFC model showing how observed convolved reward is compared with existing maps of convolved reward using Bayesian inference to determine which context is currently most likely, (C) Schematic of joint model architecture, showing how the context likelihoods generated by both HPC and PFC models are combined for contextual inference and action selection. (TIF) [file pcbi.1014093.s001.tif]

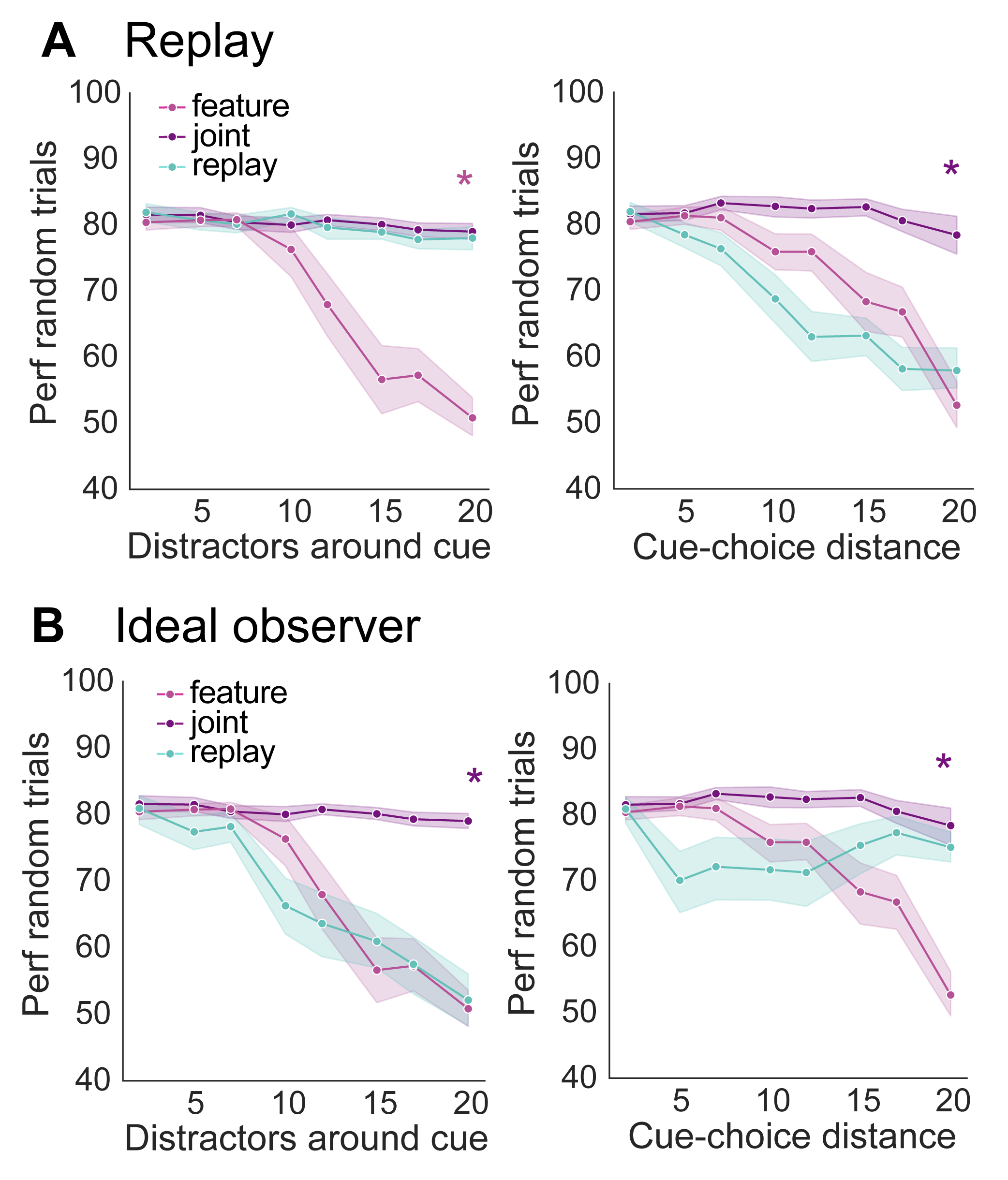

Supplement: S2 Fig — Performance on random trials with increasing distractors around cue or increasing distance from cue to choice using (A) replay of trial observations into the context map inferred by outcome inference at the end of trials, (B) replacing the outcome inference algorithm with an ideal observer model. * indicates p < 0.05, statistical results are detailed in S1 Table. (TIF) [file pcbi.1014093.s002.tif]

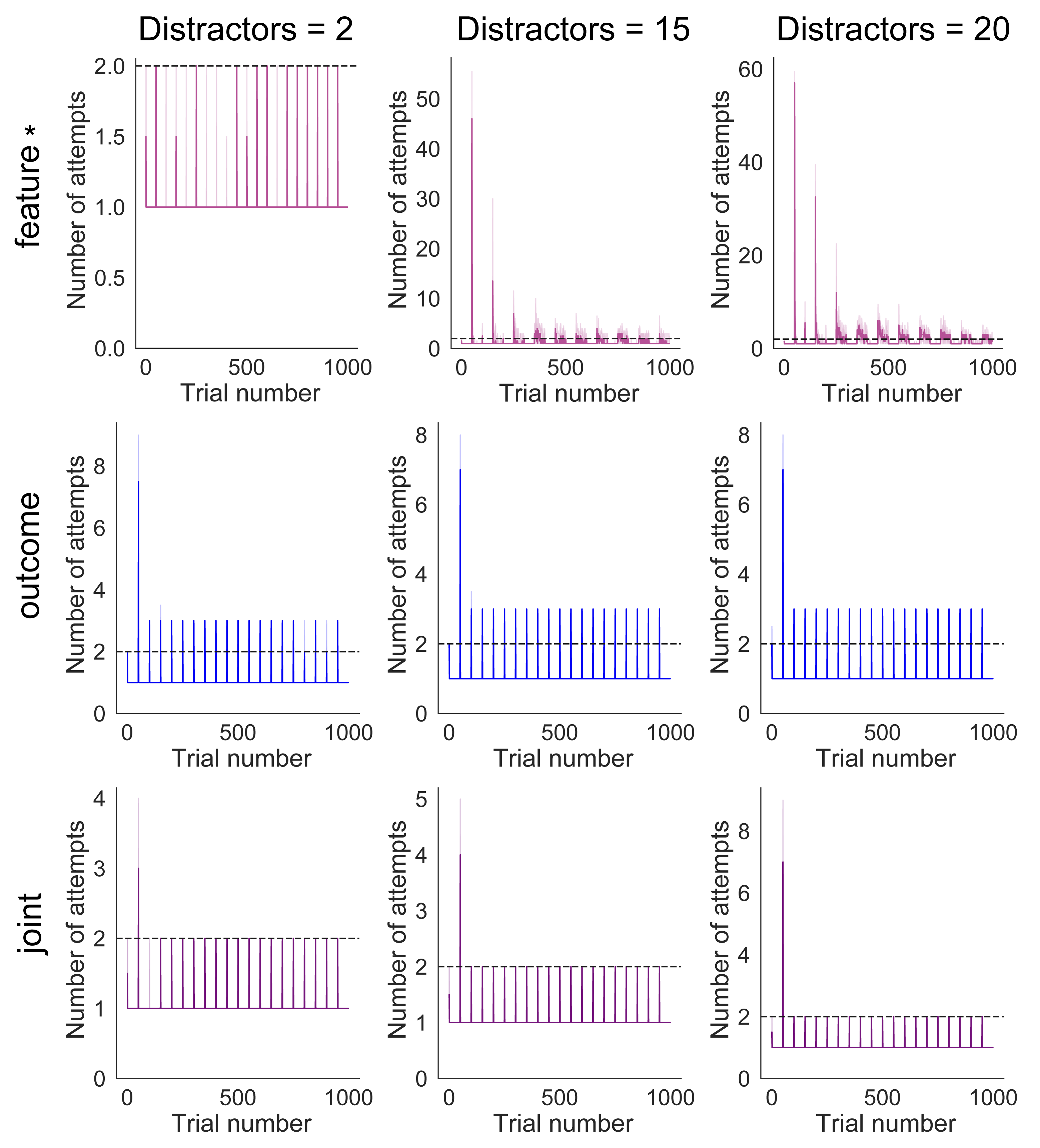

Supplement: S3 Fig — Number of attempts required to make the correct choice on each trial across blocks of trials for different numbers of distractor features (left to right) for FI, OI and joint algorithms (top to bottom). * indicates p < 0.05, statistical results are detailed in S1 Table. (TIF) [file pcbi.1014093.s003.tif]

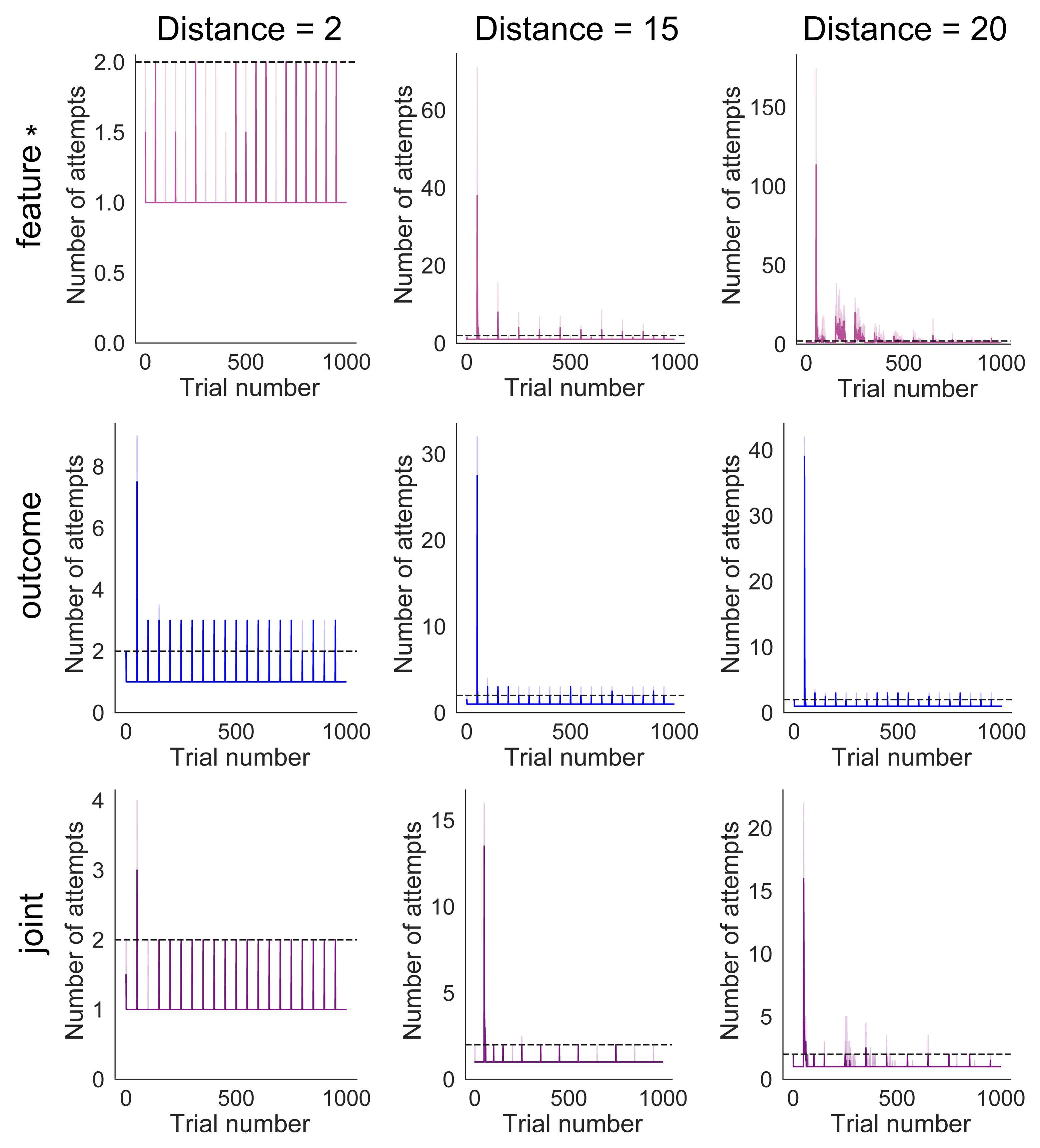

Supplement: S4 Fig — Number of attempts required to make the correct choice on each trial across blocks of trials for different cue-choice distances (left to right) for FI, OI and joint algorithms (top to bottom). * indicates p < 0.05, statistical results are detailed in S1 Table. (TIF) [file pcbi.1014093.s004.tif]

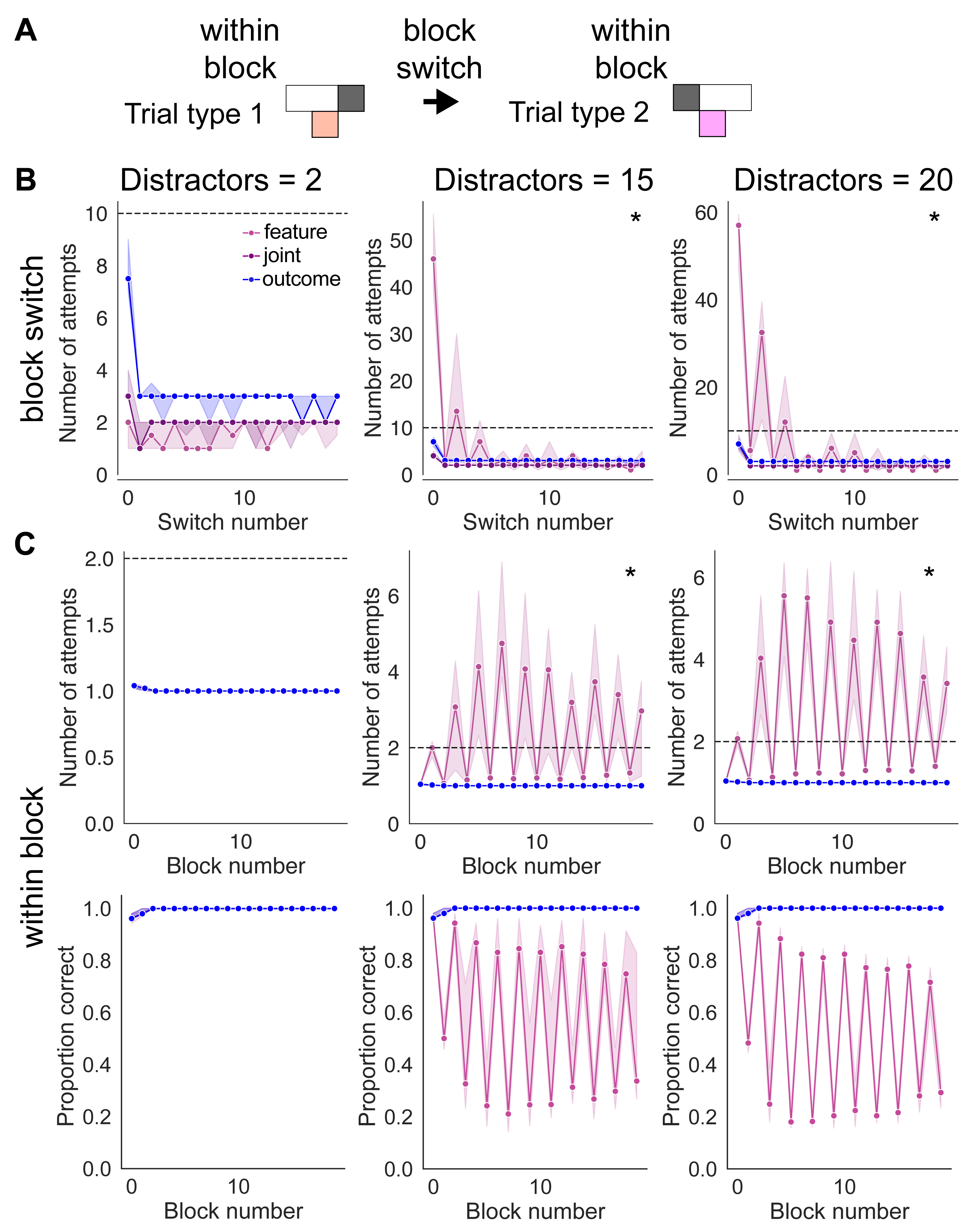

Supplement: S5 Fig — Number of attempts required to make the correct choice on each trial (A,B) directly following a block switch or (A,C) within a block for different numbers of distractor features (left to right) for FI, OI and joint algorithms (top to bottom). * indicates p < 0.05, statistical results are detailed in S1 Table. (TIF) [file pcbi.1014093.s005.tif]

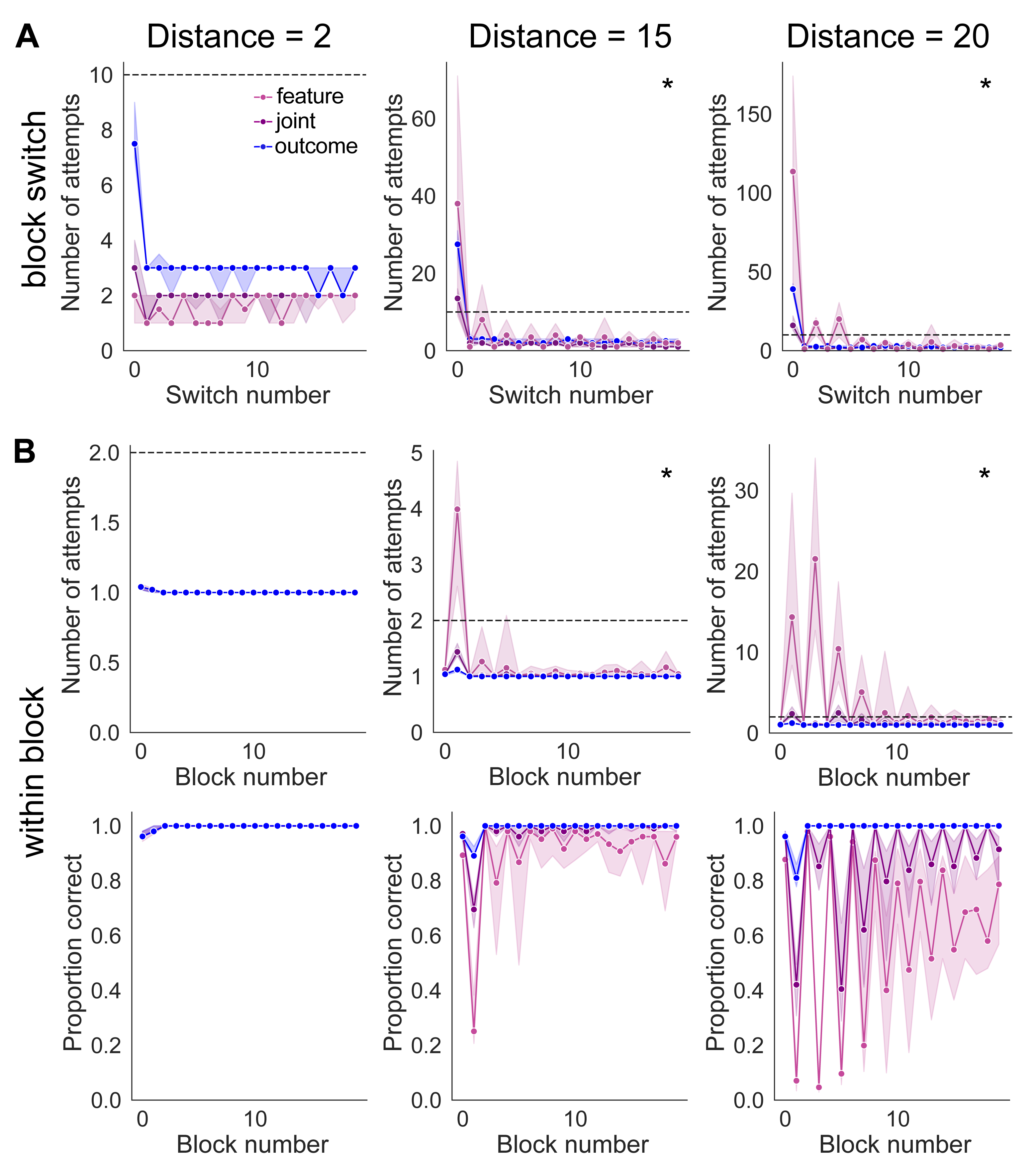

Supplement: S6 Fig — Number of attempts required to make the correct choice on each trial (A) directly following a block switch or (B) within a block for different cue-choice distances (left to right) for FI, OI and joint algorithms (top to bottom). * indicates p < 0.05, statistical results are detailed in S1 Table. (TIF) [file pcbi.1014093.s006.tif]

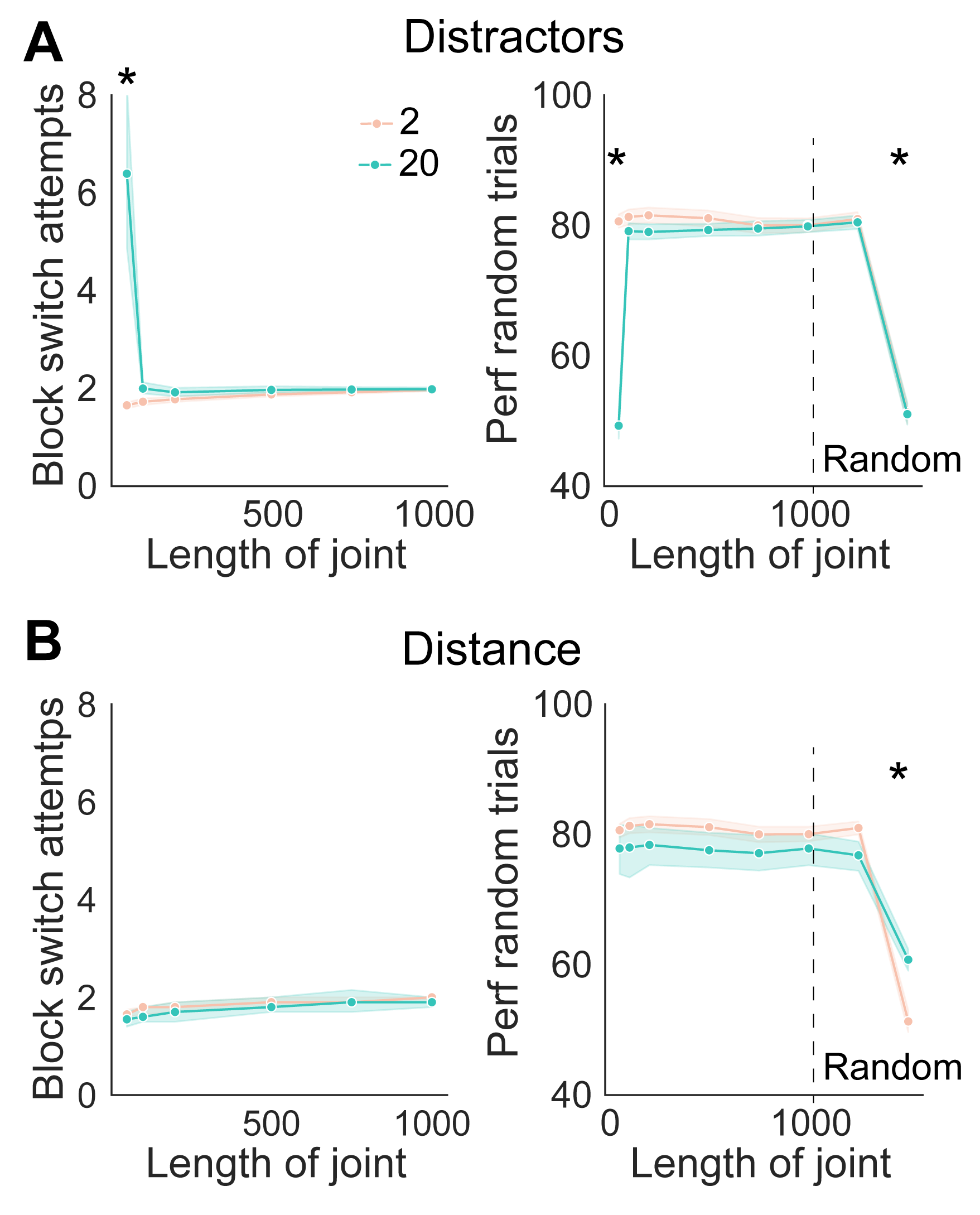

Supplement: S7 Fig — Number of attempts required on last 500 blocks of trials (left) and percent correct on last 100 random trials (right), depending on how long a joint task estimate is used for inference, for distractors around cue (A) and cue-choice distance (B). * indicates p < 0.05, statistical results are detailed in S1 Table. (TIF) [file pcbi.1014093.s007.tif]

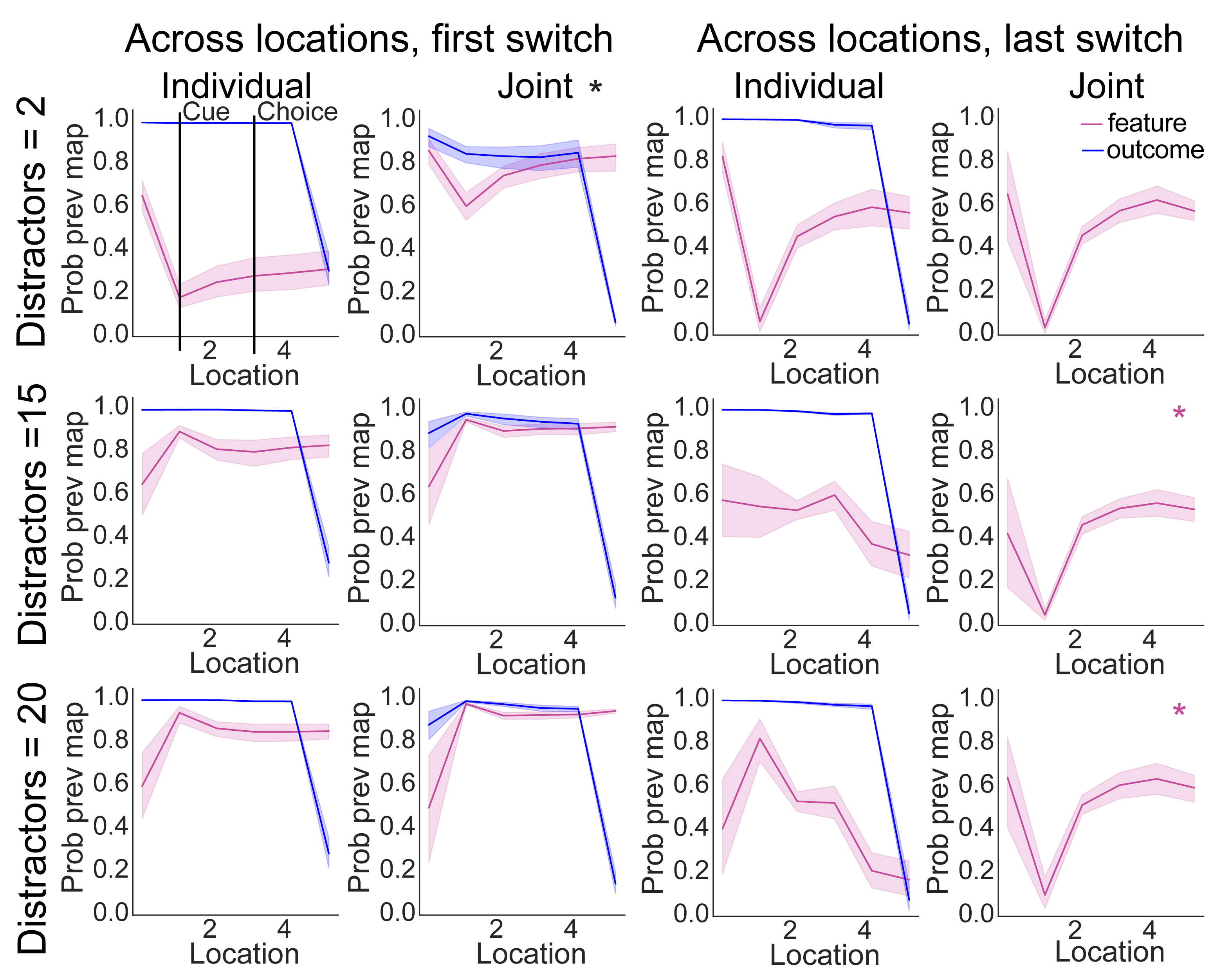

Supplement: S8 Fig — Average posterior probabilities across locations of the FI and OI maps representing the previous (now incorrect) trial type following the first attempt on the first (left) and last (right) block switch, in individual (left) and joint (right) algorithms, across increasing distractor features (top to bottom). * indicates p < 0.05, statistical results are detailed in S1 Table. (TIF) [file pcbi.1014093.s008.tif]

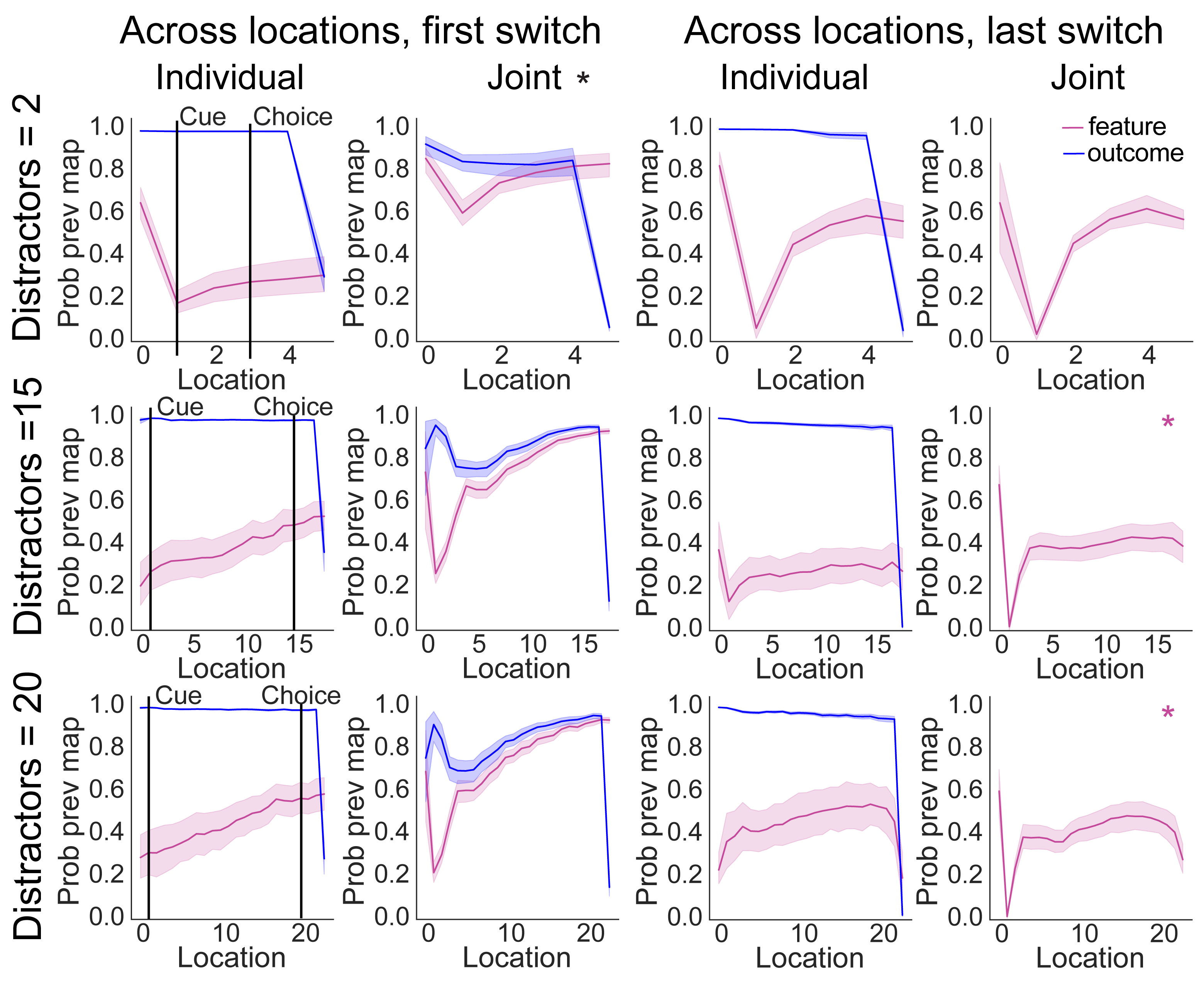

Supplement: S9 Fig — Average posterior probabilities across locations of the FI and OI maps representing the previous (now incorrect) trial type following the first attempt on the first (left) and last (right) block switch, in individual (left) and joint (right) algorithms, across increasing cue-choice distances (top to bottom). * indicates p < 0.05, statistical results are detailed in S1 Table. (TIF) [file pcbi.1014093.s009.tif]

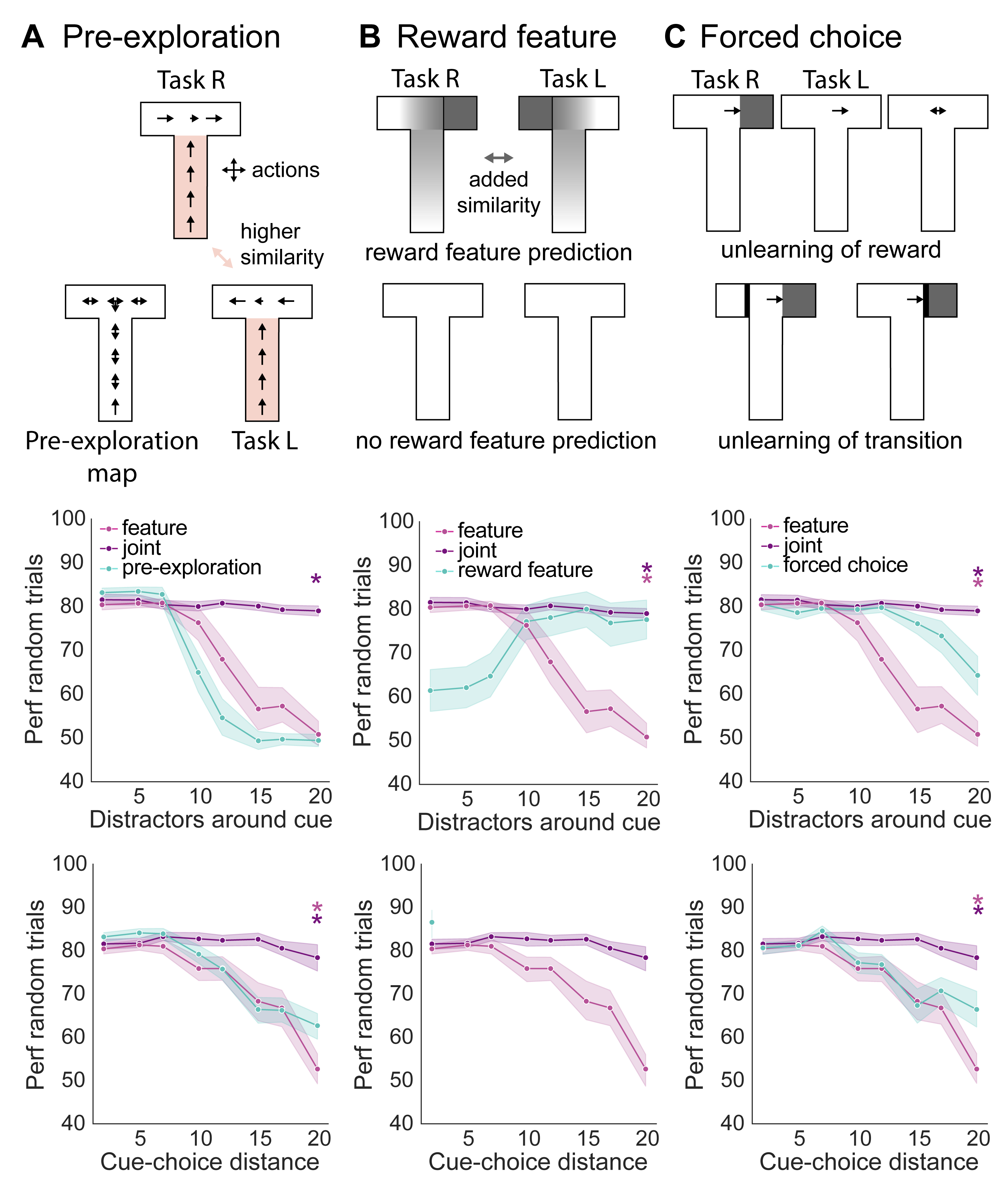

Supplement: S10 Fig — Schematics of effects on performance (top) and effects on performance on random trials (bottom) with increasing distractors around cue or increasing distance from cue to choice using (A) pre-exploration of the environment without rewards or predictive cues, (B) an additional feature that indicates reward presence (the data in cue-choice distance is incomplete as individual agents took over the 48hr time limit to run), (C) forced choice where the incorrect arm is blocked off during learning. * indicates p < 0.05, statistical results are detailed in S1 Table. (TIF) [file pcbi.1014093.s010.tif]

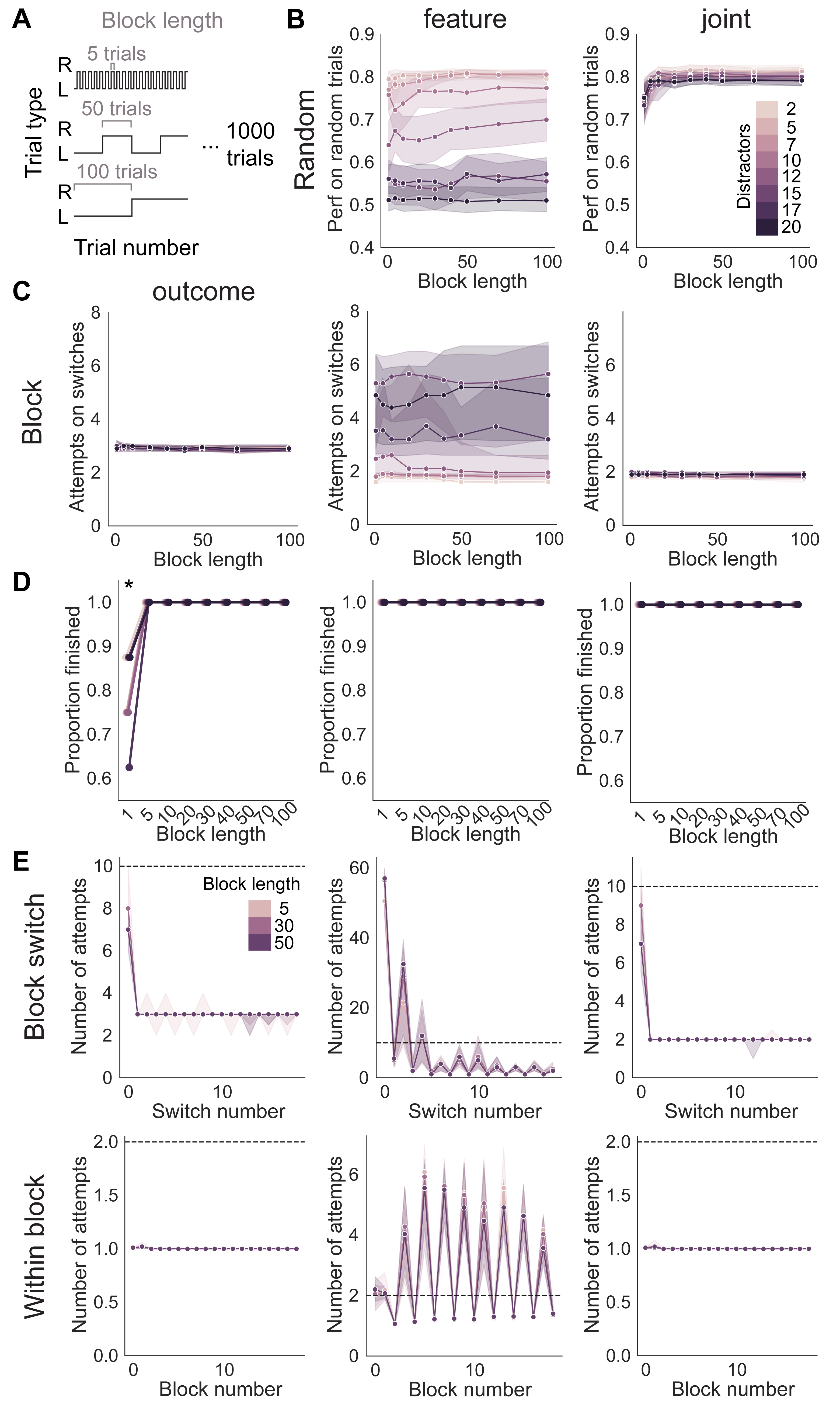

Supplement: S11 Fig — (A) Schematic of different block lengths tested, (B) Performance on random trials and (C) block trials with increasing distractor features depending on block length, (D) Proportion of agents that completed the task within the runtime limit (48hrs per 5 agents), (E) Number of attempts required to make the correct choice on each trial directly following a block switch (top) or within a block (bottom) for 20 distractors. * indicates p < 0.05, statistical results are detailed in S1 Table. (TIF) [file pcbi.1014093.s011.tif]

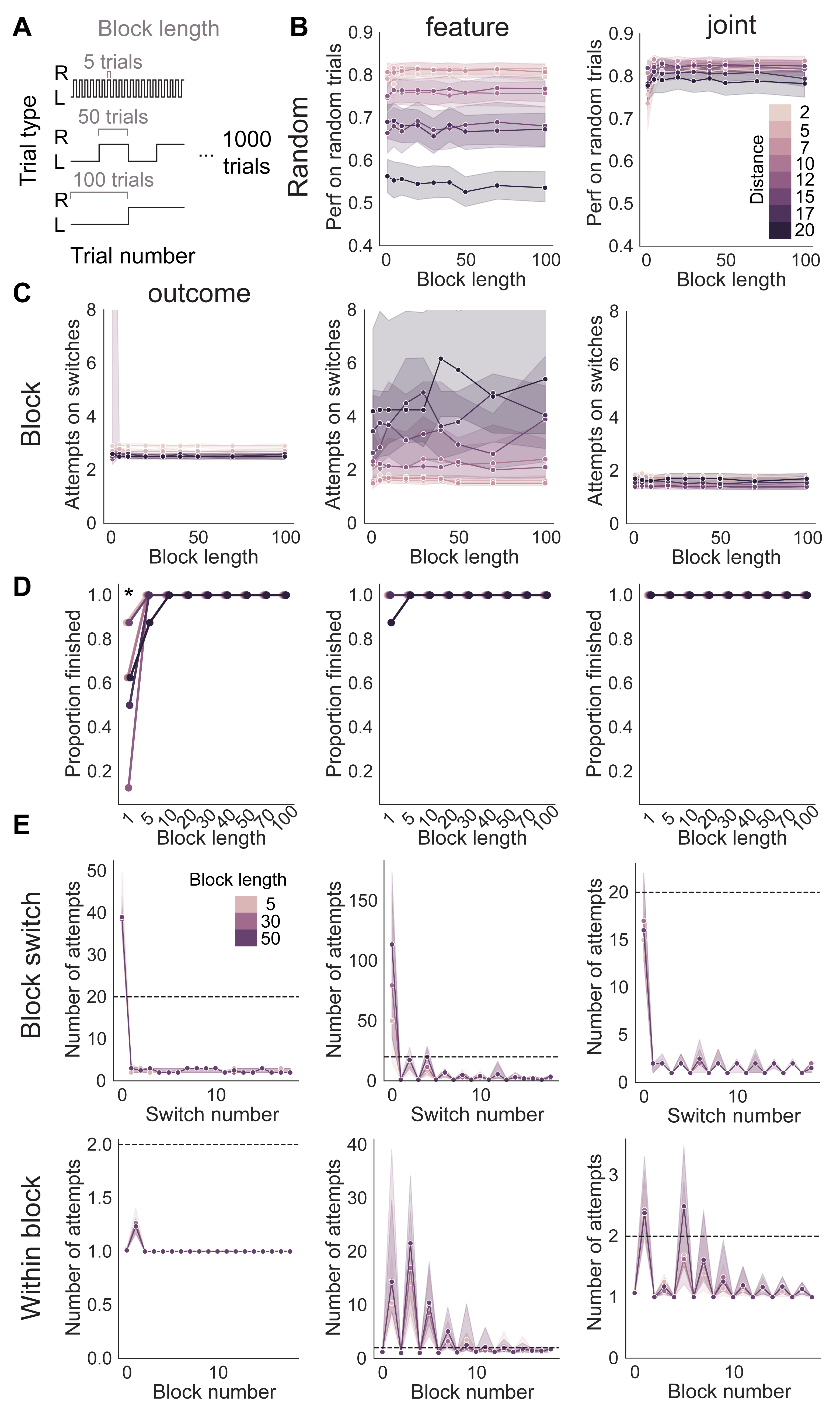

Supplement: S12 Fig — (A) Schematic of different block lengths tested, (B) Performance on random trials and (C) block trials with increasing cue-choice distance depending on block length, (D) Proportion of agents that completed the task within the runtime limit (48hrs per 5 agents), (E) Number of attempts required to make the correct choice on each trial directly following a block switch (top) or within a block (bottom) for cue-choice distance 20. * indicates p < 0.05, statistical results are detailed in S1 Table. (TIF) [file pcbi.1014093.s012.tif]

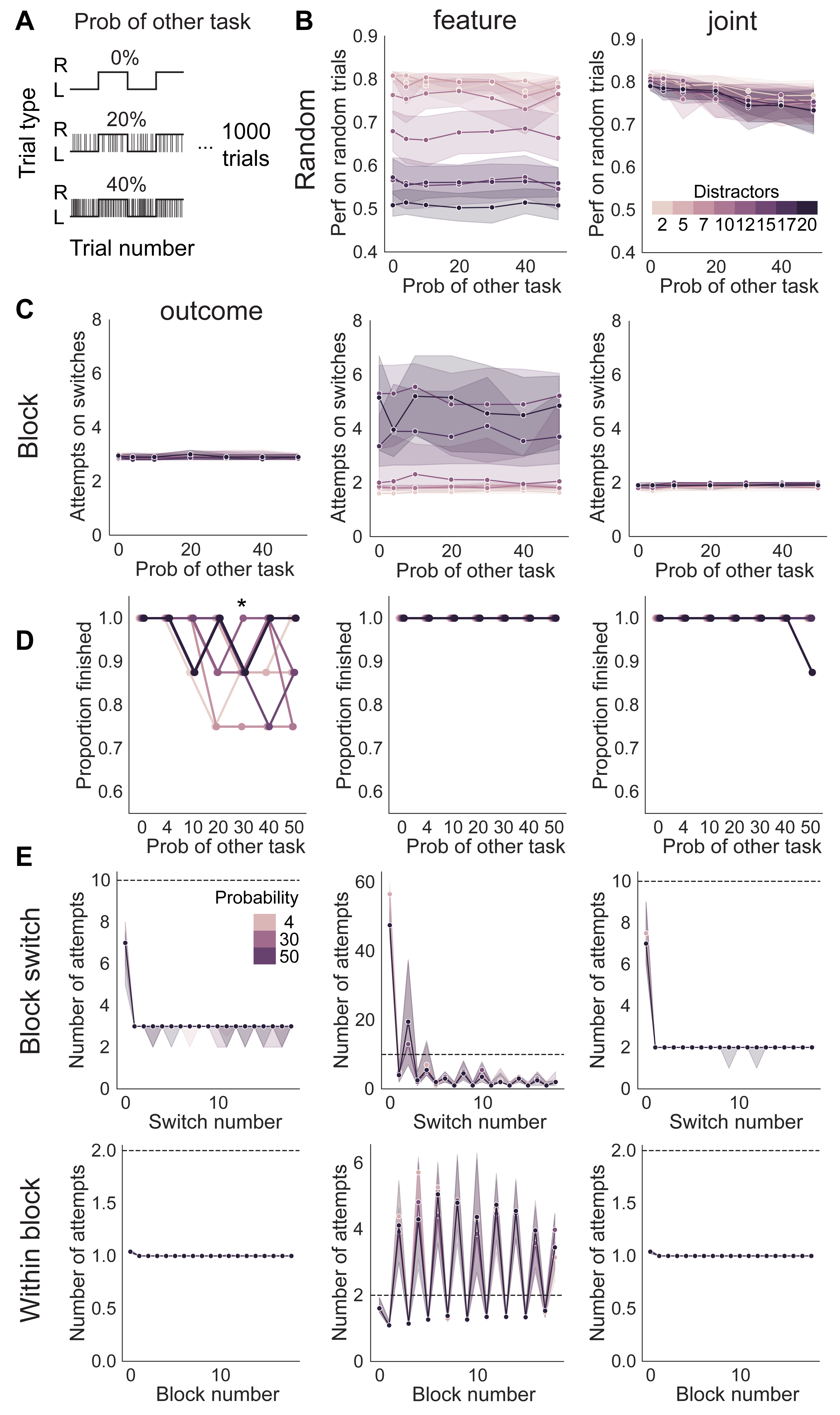

Supplement: S13 Fig — (A) Schematic of different probabilistic setups tested, (B) Performance on random trials and (C) block trials with increasing distractor features depending on block probabilities, (D) Proportion of agents that completed the task within the runtime limit (48hrs per 5 agents), (E) Number of attempts required to make the correct choice on each trial directly following a block switch (top) or within a block (bottom) for 20 distractors. * indicates p < 0.05, statistical results are detailed in S1 Table. (TIF) [file pcbi.1014093.s013.tif]

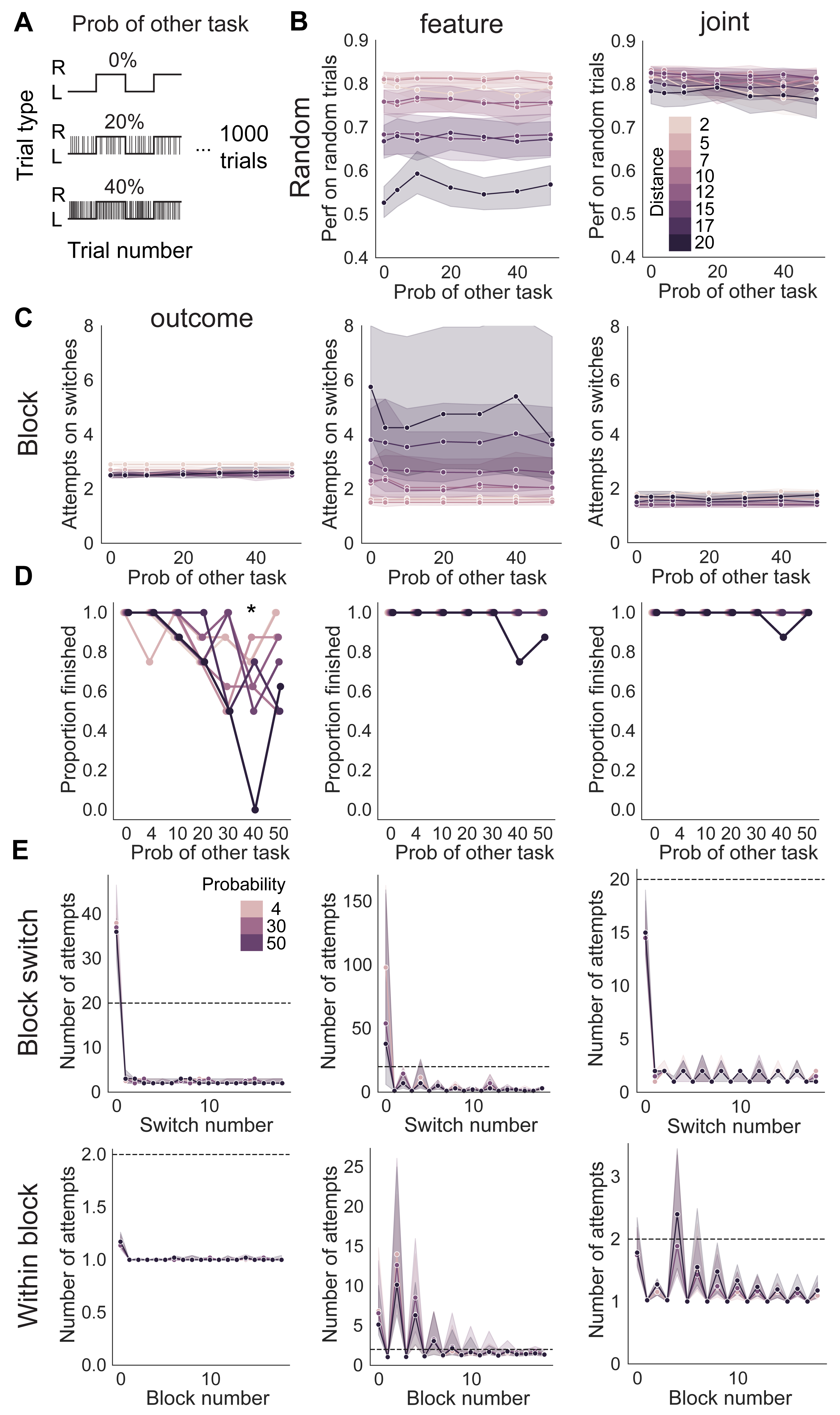

Supplement: S14 Fig — (A) Schematic of different probabilistic setups tested, (B) Performance on random trials and (C) block trials with increasing cue-choice distance depending on block probabilities, (D) Proportion of agents that completed the task within the runtime limit (48hrs per 5 agents), (E) Number of attempts required to make the correct choice on each trial directly following a block switch (top) or within a block (bottom) for cue-choice distance 20. * indicates p < 0.05, statistical results are detailed in S1 Table. (TIF) [file pcbi.1014093.s014.tif]

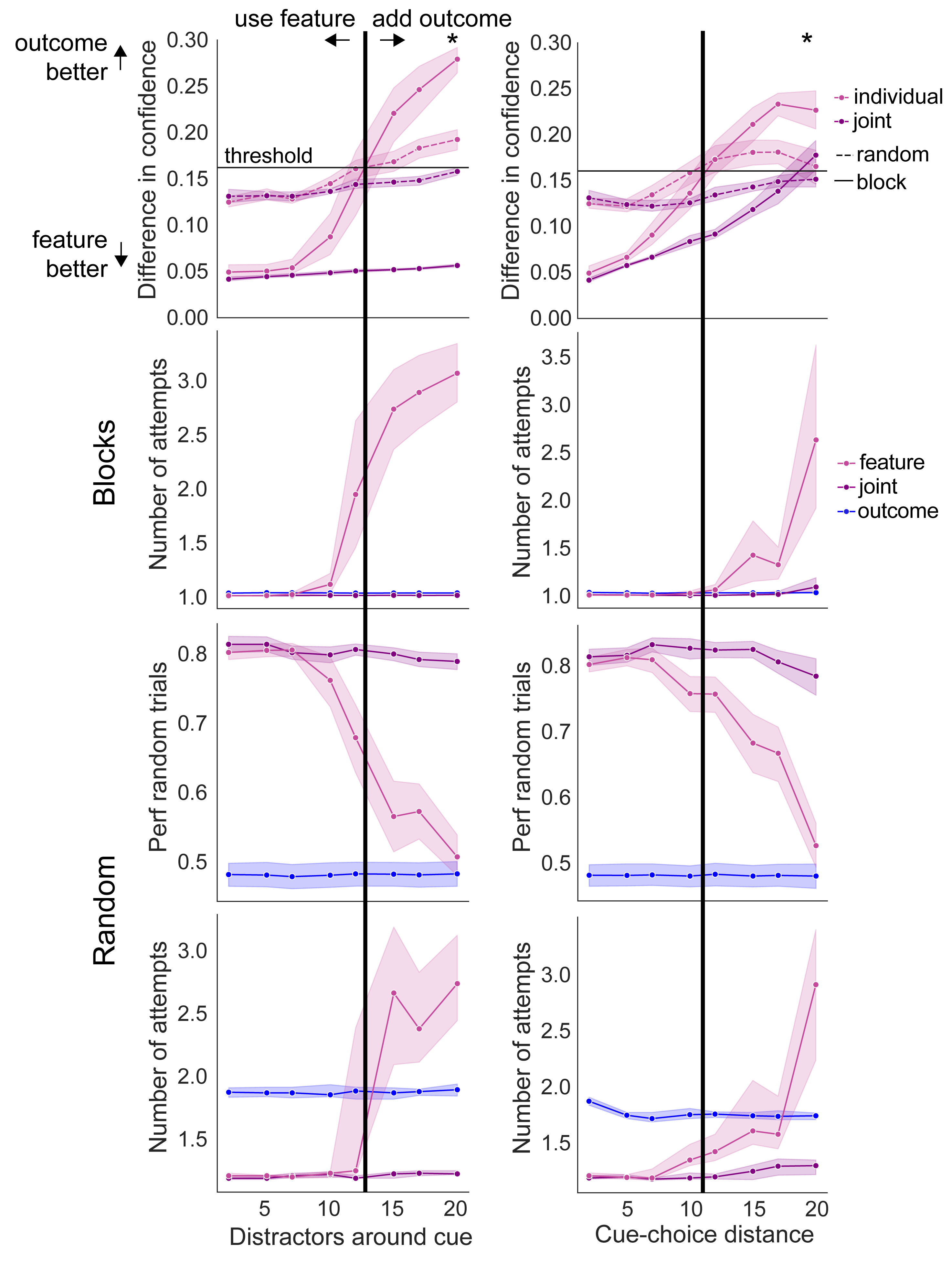

Supplement: S15 Fig — Difference in confidence between outcome inference estimate and feature inference estimate across random and block trials, allowing for thresholding when to use joint inference and when to use feature inference (top). Number of attempts on blocks of trials (middle) and number of attempts and performance on random trials (bottom) across increasing distractors (left) and cue-choice distance (right), showing when the selected threshold would result in use of joint inference rather than feature inference. * indicates p < 0.05, statistical results are detailed in S1 Table. (TIF) [file pcbi.1014093.s015.tif]

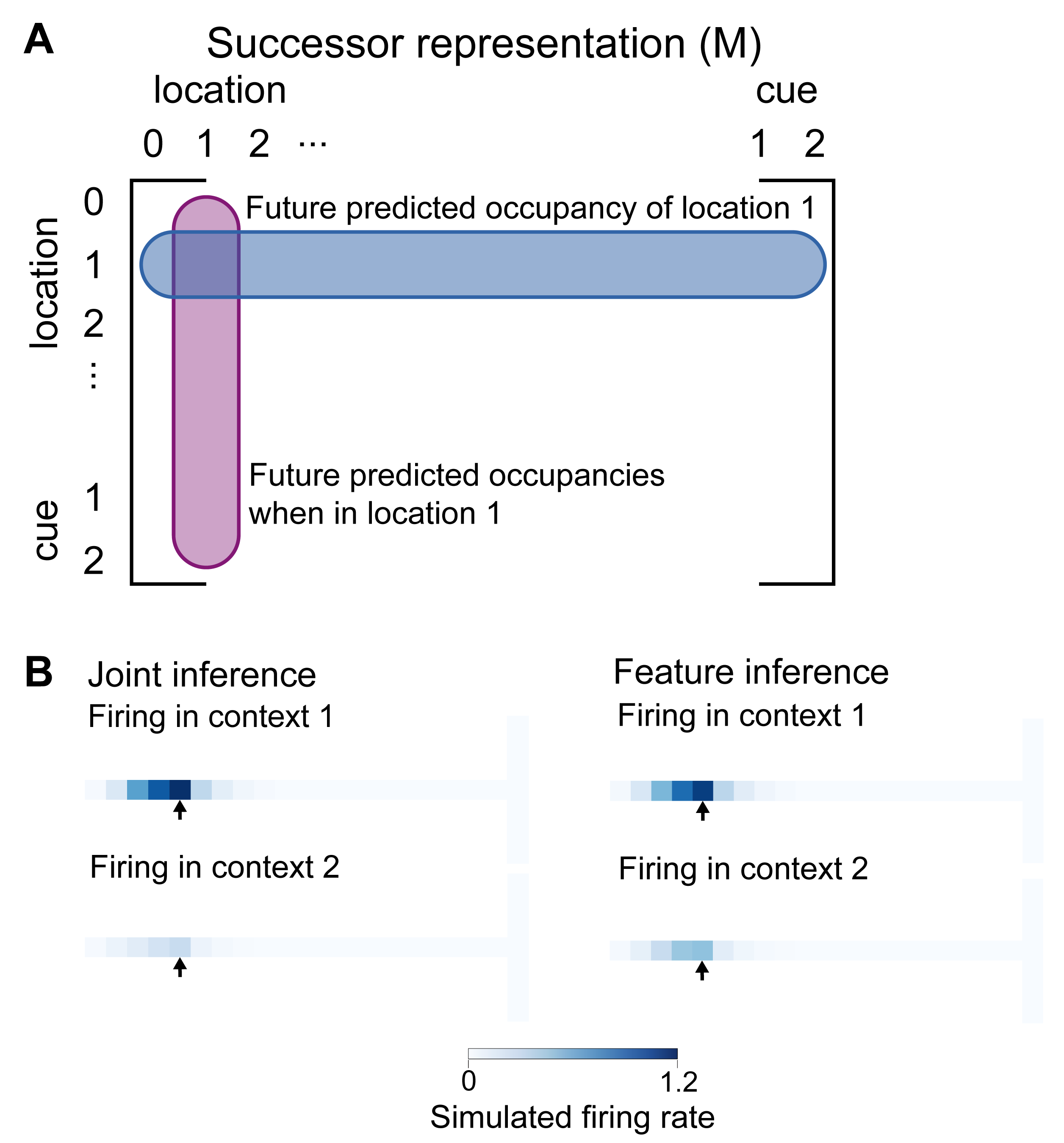

Supplement: S16 Fig — (A) Schematic showing the distinction in plots of successor representations used in Figs 3 and 6 to investigate the representations learnt by agents looking at the predicted future occupancy of all other features given the current location (pink) and the plots used to show simulated firing rates in Fig 7, which represent the future predicted occupancy of a specific location across all other features (blue), (B) Simulated firing rate maps from Fig 7A originally plotted using the traditional colormap for place cells, allowing for direct comparison with [35], in a single sequential colormap instead. (TIF) [file pcbi.1014093.s016.tif]
