# Supplementary material for: Contextual inference through flexible integration of environmental features and behavioural outcomes
Source: PLoS Comput Biol. 2026 Mar 20;22(3):e1014093. doi: 10.1371/journal.pcbi.1014093 (PMC13029755; doi:10.1371/journal.pcbi.1014093)
Supplement: S1 Table — (PDF) [file pcbi.1014093.s017.pdf]

Table 1: Statistical results

Figure	Test	Result	Post-hoc test	Result
3D left	Welch ANOVA	$F_{(4,92)} = 104$, p < 0.001	Games-Howell	FI-OI: $t_{(68)}$=-17, p < 0.001; FI-SR1: $t_{(77)}$ = -14, p < 0.001; FI-SR: $t_{(39)}$ = -9, p < 0.001; OI-SR: $t_{(39)}$ = -10, p < 0.001
3D right	ANOVA	$F_{(4,195)} = 330$, p < 0.001	Tukey	FI-OI $t_{(39)}$ = 18, p < 0.001; FI-SR $t_{(39)}$ = 18, p < 0.001; FI-SR1 $t_{(39)}$ = 18, p < 0.001; FI-TD $t_{(39)}$ = 17, p < 0.001
4C left	Mixed ANOVA	interaction $F_{(7,546)} = 11$, p < 0.001 algorithm $F_{(1,78)} = 13$, p < 0.001	post-hoc t-tests with BHF	For interaction: p < 0.05 for all except distractors = 10
			Linear regression	FI interaction distractors*attempts: R^2 = 0.15, slope = 0.3, x-intercept = 0.6, $p_{(318)} < 0.001$
4C right	Mixed ANOVA	interaction $F_{(7,546)} = 5$, p < 0.001 algorithm $F_{(1,78)} = 15$, p < 0.001	post-hoc t-tests BHF	For interaction: p < 0.05 for all except distance = 10
			Linear regression	FI interaction cue-choice distance*attempts, R^2 = 0.05, slope = 0.4, x-intercept = 0.02, $p_{(318)} < 0.001$
4D left	Mixed ANOVA	interaction $F_{(7,546)} = 55$, p < 0.001, algorithm $F_{(1,78)} = 309$, p < 0.001	post-hoc t-tests with BHF	For interaction: p < 0.05 for all except distractors = 20
			Linear regression	FI interaction distractors*performance, R^2 = 0.5, slope = -2, x-intercept = 89, $p_{(318)} < 0.001$
4D right	Mixed ANOVA	interaction $F_{(7,546)} = 42$, p < 0.001, algorithm $F_{(1,78)} = 524$, p < 0.001	post-hoc t-tests BHF	Interaction: p < 0.05 for all
			Linear regression	FI interaction cue-choice distance*attempts, R^2 = 0.4, slope = -1, x-intercept = 90, $p_{(318)} < 0.001$

Continued on next page

Table 1: Statistical results (Continued)

5C left	Mixed ANOVA	interaction $F_{(14,819)} = 47$, $p < 0.001$, algorithm $F_{(2,117)} = 544$, $p < 0.001$	post-hoc t-tests with BHF	Algorithms: $p < 0.05$ for all
5C right	Mixed ANOVA	interaction $F_{(14,819)} = 30$, $p < 0.001$, algorithm $F_{(2,117)} = 711$, $p < 0.001$	post-hoc t-tests BHF	Algorithms: $p < 0.05$ for all
5D left	Mixed ANOVA	interaction $F_{(14,819)} = 10$, $p < 0.001$, algorithm $F_{(2,117)} = 47$, $p < 0.001$	post-hoc t-tests with BHF	Algorithms: $p < 0.05$ for all
5D right	Mixed ANOVA	interaction $F_{(14,819)} = 5$, $p < 0.001$, algorithm $F_{(2,117)} =23$, $p < 0.001$	post-hoc t-tests BHF	Algorithms: $p < 0.05$ for all
6A top	Mixed ANOVA	interaction distractors*algorithm: $F_{(14,819)} = 58$, $p < 0.001$		
6A bottom	Mixed ANOVA	interaction distance*algorithm: $F_{(14,819)} = 4$, $p < 0.001$		
6C top	Mixed ANOVA	distractors*algorithm: correct cue $F_{(7,546)} = 57$, $p < 0.001$, incorrect cue $F_{(7,546)} = 47$, $p < 0.001$		
6C bottom	Mixed ANOVA	interaction distance*algorithm: correct cue $F_{(7,546)} = 9$, $p < 0.001$, incorrect cue $F_{(7,546)} = 6$, $p < 0.001$		
6D top	t-test	$t_{(40)} = -12$, $p < 0.001$		
6D bottom	Mixed ANOVA	interaction distance*algorithm: $F_{(18,1224)} = 7$, $p < 0.001$		
7B left	Paired t-test	area under the curve of simulated firing rate on preferred vs non-preferred trials, $t_{(39)} = 25$, $p < 0.001$		
7B right	Paired t-test	area under the curve of simulated firing rate on preferred vs non-preferred trials, $t_{(39)} = 8$, $p < 0.001$		

Continued on next page

Table 1: Statistical results (Continued)

7B	Paired t-test	Joint vs feature: difference in area under the curve of simulated firing rate on preferred vs non-preferred trials, $t_{(78)} = 7$, p < 0.001		
7C	Mixed ANOVA	interaction distance from cue*difference in splitter probabilities: $F_{(17,1326)} = 8$, p < 0.001		
7E left	Mixed ANOVA	interaction difference in splitter probabilities*algorithm: $F_{(7,546)} = 61$, p < 0.001		
7E right	Linear regression	interaction performance on random trials*difference in splitter probabilities, $R^2 = 0.78$, slope = 0.01, x-intercept = -0.5, $p_{(318)} < 0.001$		
7F left	Mixed ANOVA	interaction difference in splitter probabilities*algorithm: $F_{(7,546)} = 22$, p < 0.001		
7F right	Linear regression	interaction performance on random trials*difference in splitter probabilities, $R^2 = 0.25$, slope = 0.006, x-intercept = 0.13, $p_{(318)} < 0.001$		
8B	T-test	$t_{(78)} = 5$, p < 0.001		
8C	Mixed ANOVA	algorithm $F_{(1,78)} = 10$, p = 0.002; interaction $F_{(4,312)} = 4$, p = 0.006	post-hoc t-tests with BHF	Interaction: p < 0.05 for 0-100 and 100-200
8E	Mann-Whitney U	U = 1558, p < 0.001		
8F	Mixed ANOVA	algorithm $F_{(1,78)} = 73$, p < 0.001; interaction $F_{(4,312)} = 0.5$, p = 0.8		
S2A left	Mixed ANOVA	algorithm $F_{(2,117)} = 155$, p < 0.001	post-hoc t-tests with BHF	Algorithms: p < 0.05 for feature-replay

Continued on next page

Table 1: Statistical results (Continued)

S2A right	Mixed ANOVA	algorithm $F_{(2,117)} = 142$, p < 0.001	post-hoc t-tests with BHF	Algorithms: p $<$ 0.05 for all
S2B left	Mixed ANOVA	algorithm $F_{(2,117)} = 133$, p < 0.001	post-hoc t-tests with BHF	Algorithms: p $<$ 0.05 for joint-ideal
S2B right	Mixed ANOVA	algorithm $F_{(2,117)} = 60$, p < 0.001	post-hoc t-tests with BHF	Algorithms: p $<$ 0.05 for joint-ideal
S3	Mixed ANOVA	algorithm $F_{(2,117)} = 107$, p < 0.01	post-hoc t-tests with BHF	Algorithms: p $<$ 0.001 for feature-joint and feature-outcome
S4	Mixed ANOVA	algorithm $F_{(2,117)} = 15$, p < 0.01	post-hoc t-tests with BHF	Algorithms: p $<$ 0.005 for feature-joint and feature-outcome
S5B	Mixed ANOVA	distractors 15: algorithm $F_{(2,117)} = 47$, p < 0.001; interaction switch number*algorithm $F_{(36,2106)} = 8$, p < 0.001; distractors 20: algorithm $F_{(2,117)} = 141$, p < 0.001; interaction switch number*algorithm $F_{(36,2106)} = 18$, p < 0.001	post-hoc t-tests with BHF	Algorithms: p $<$ 0.05 for all in distractors 15 and 20
	Mixed ANOVA	interaction number distractors*algorithm: $F_{(4,234)} = 3$, p $= 0.03$	post-hoc t-tests with BHF	Interaction: p $<$ 0.05 for distractors 15 and 20
S5C	Mixed ANOVA	distractors 2: algorithm $F_{(2,117)} = 4$, p $= 0.03$; interaction block number*algorithm $F_{(38,2223)} = 2$, p < 0.001; distractors 15: algorithm $F_{(2,117)} = 80$, p < 0.001; interaction block number*algorithm $F_{(38,2223)} = 18$, p < 0.001; 20: algorithm $F_{(2,117)} = 264$, p < 0.001; interaction block number*algorithm $F_{(38,2223)} = 32$, p < 0.001	post-hoc t-tests with BHF	Algorithms: p $<$ 0.05 for all for distractors 15/20 for joint-others for distractors 2

Continued on next page

Table 1: Statistical results (Continued)

	Mixed ANOVA	algorithm $F_{(2,117)} = 81$, p < 0.001; interaction number distractors*algorithm $F_{(4,234)} = 181$, p < 0.001	post-hoc t-tests with BHF	Algorithm: p < 0.001 for feature-others
S6A	Mixed ANOVA	distance 15: interaction switch number*algorithm: $F_{(36,2106)} = 2$, p $= 0.002$; distance 20: algorithm $F_{(2,117)} = 8$, p < 0.001; interaction switch number*algorithm $F_{(36,2106)} = 4$, p < 0.001	post-hoc t-tests with BHF	Algorithms: distance 20: p < 0.05 for feature-joint and feature-outcome; Interaction: distance 15: p < 0.05 outcome-joint all, feature-outcome switch 2, feature-joint switch 1,3
	Mixed ANOVA	interaction number distance*algorithm $F_{(4,234)} =$ 3, p $= 0.02$	post-hoc t-tests with BHF	Interaction: p < 0.05 for distance 15/20: feature-joint and 20: feature-outcome
S6B	Mixed ANOVA	distance 15: algorithm $F_{(2,117)} = 10$, p < 0.001; interaction block number*algorithm $F_{(38,2223)} =$ 3, p < 0.001; 20: algorithm $F_{(2,117)} = 11$, p < 0.001; interaction block number*algorithm $F_{(38,2223)} = 5$, p < 0.001	post-hoc t-tests with BHF	Interaction: p < 0.05 for joint-others for distance 15/20
	Mixed ANOVA	algorithm $F_{(2,117)} = 5$, p < 0.001; interaction number distance*algorithm $F_{(4,234)} = 3$, p < 0.001	post-hoc t-tests with BHF	Algorithm: p < 0.001 for feature-joint
S7A left	Mixed ANOVA	number of distractors $F_{(1,78)} = 28$, p < 0.001; length of joint $F_{(5,390)} =$ 19, p < 0.001; interaction $F_{(5,390)} = 23$, p < 0.001	post-hoc t-tests with BHF	p < 0.05 for all except length of joint 100-200,500, 750-500,1000 and interaction 750,1000
S7A right	Mixed ANOVA	number of distractors $F_{(1,38)} = 60$, p < 0.001; length of joint $F_{(7,277)} =$ 277, p < 0.001; interaction $F_{(7,266)} = 77$, p < 0.001	post-hoc t-tests with BHF	p < 0.05 for length of joint 1500-others, 50-others and interaction length of joint 50
S7B left	Mixed ANOVA	length of joint $F_{(5,390)} = 2$, p $= 0.046$	post-hoc t-tests with BHF	p > 0.05 for all

Continued on next page

Table 1: Statistical results (Continued)

S7B right	Mixed ANOVA	length of joint $F_{(7,226)} = 74$, $p < 0.001$; interaction length of joint*distance $F_{(7,226)} = 6$, $p < 0.001$	post-hoc t-tests with BHF	$p < 0.05$ for length of joint 1500-others and interaction at 1500
S8 left 2 feature	Mixed ANOVA	individual/joint $F_{(1,73)} = 136$, $p < 0.001$; interaction locations*individual/joint $F_{(5,365)} = 33$, $p < 0.001$	post-hoc t-tests with BHF	$p < 0.001$ for all locations
S8 left 15 feature	Mixed ANOVA	interaction locations*individual/joint $F_{(5,130)} = 2$, $p = 0.045$	post-hoc t-tests with BHF	$p < 0.05$ for location 1
S8 left 20 feature	Mixed ANOVA	interaction locations*individual/joint $F_{(5,95)} = 17$, $p = 0.042$	post-hoc t-tests with BHF	$p < 0.05$ for location 5
S8 left 2 outcome	Mixed ANOVA	individual/joint $F_{(1,73)} = 45$, $p < 0.001$; interaction locations*individual/joint $F_{(5,365)} = 7$, $p < 0.001$	post-hoc t-tests with BHF	$p < 0.05$ for all except location 0
S8 left 15 outcome	Mixed ANOVA	individual/joint $F_{(1,28)} = 23$, $p < 0.001$; interaction locations*individual/joint $F_{(5,140)} = 2$, $p = 0.001$	post-hoc t-tests with BHF	$p < 0.05$ for locations 3,5
S8 left 20 outcome	Mixed ANOVA	individual/joint $F_{(1,23)} = 43$, $p < 0.001$; interaction locations*individual/joint $F_{(5,115)} = 5$, $p < 0.001$	post-hoc t-tests with BHF	$p < 0.05$ for locations 1,2,5
S8 right 2 feature	Mixed ANOVA	interaction locations*individual/joint $F_{(5,125)} = 3$, $p = 0.01$	post-hoc t-tests with BHF	$p > 0.05$ for all
S8 right 15 feature	Mixed ANOVA	individual/joint $F_{(1,14)} = 5$, $p = 0.03$		
S8 right 20 feature	Mixed ANOVA	interaction locations*individual/joint $F_{(5,100)} = 6$, $p < 0.001$	post-hoc t-tests with BHF	$p < 0.05$ for location 1
S9 left 2 feature	Mixed ANOVA	individual/joint $F_{(1,73)} = 136$, $p < 0.001$; interaction locations*individual/joint $F_{(5,365)} = 33$, $p < 0.001$	post-hoc t-tests with BHF	$p < 0.001$ for all locations

Continued on next page

Table 1: Statistical results (Continued)

S9 left 15 feature	Mixed ANOVA	individual/joint $F_{(1,31)} = 33$, $p < 0.001$; interaction locations*individual/joint $F_{(18,558)} = 4$, $p < 0.001$	post-hoc t-tests with BHF	$p < 0.05$ for all except locations 1,2
S9 left 20 feature	Mixed ANOVA	individual/joint $F_{(1,45)} = 13$, $p < 0.001$; interaction locations*individual/joint $F_{(23,1035)} = 6$, $p < 0.001$	post-hoc t-tests with BHF	$p < 0.05$ for all except locations 0-7
S9 left 2 outcome	Mixed ANOVA	individual/joint $F_{(1,73)} = 45$, $p < 0.001$; interaction locations*individual/joint $F_{(5,365)} = 7$, $p < 0.001$	post-hoc t-tests with BHF	$p < 0.05$ for all except location 0
S9 left 15 outcome	Mixed ANOVA	individual/joint $F_{(1,13)} = 12$, $p = 0.004$; interaction locations*individual/joint $F_{(18,234)} = 5$, $p < 0.001$	post-hoc t-tests with BHF	$p < 0.05$ for location 10
S9 left 20 outcome	Mixed ANOVA	individual/joint $F_{(1,18)} = 12$, $p = 0.003$; interaction locations*individual/joint $F_{(23,414)} = 7$, $p < 0.001$	post-hoc t-tests with BHF	$p < 0.05$ for locations 2-8, 10-12
S9 right 2 feature	Mixed ANOVA	interaction locations*individual/joint $F_{(5,125)} = 3$, $p = 0.01$	post-hoc t-tests with BHF	$p > 0.05$ for all
S9 right 15 feature	Mixed ANOVA	individual/joint $F_{(1,49)} = 8$, $p = 0.007$; interaction locations*individual/joint $F_{(18,882)} = 3$, $p < 0.001$	post-hoc t-tests with BHF	$p < 0.05$ for location 0,3,4
S9 right 20 feature	Mixed ANOVA	individual/joint $F_{(1,48)} = 7$, $p = 0.01$; interaction locations*individual/joint $F_{(23,1104)} = 33$, $p < 0.001$	post-hoc t-tests with BHF	$p < 0.05$ for location 1,2
S10A top	Mixed ANOVA	algorithm $F_{(2,117)} = 204$, $p < 0.001$	post-hoc t-tests with BHF	Algorithms: $p < 0.05$ for explore-joint
S10A bottom	Mixed ANOVA	algorithm $F_{(2,117)} = 90$, $p < 0.001$	post-hoc t-tests with BHF	Algorithms: $p < 0.05$ for all
S10B top	Mixed ANOVA	algorithm $F_{(2,117)} = 63$, $p < 0.001$	post-hoc t-tests with BHF	Algorithms: $p < 0.05$ for all

Continued on next page

Table 1: Statistical results (Continued)

S10C top	Mixed ANOVA	algorithm $F_{(2,117)} = 97$, p < 0.001	post-hoc t-tests with BHF	Algorithms: $p < 0.05$ for all
S10C bottom	Mixed ANOVA	algorithm $F_{(2,117)} = 72$, p < 0.001	post-hoc t-tests with BHF	Algorithms: $p < 0.05$ for all
S11B	Mixed ANOVA for block length and number distractors	joint: block length $F_{(8,351)} = 6$, $p < 0.001$	post-hoc t-tests with BHF	block length $p > 0.05$ for all
S11C	Mixed ANOVA for block length and number distractors	joint: block length $F_{(8,351)} = 5$, $p < 0.001$; outcome: block length $F_{(8,326)} = 3$, $p = 0.004$	post-hoc t-tests with BHF	block length $p > 0.05$ for all
S11D	ANOVA for block length	outcome: block length $F_{(8,63)} = 49$, $p < 0.001$	post-hoc Tukey	Outcome: block length p < 0.001 for 1-others
S11E top	Mixed ANOVA for block length and switch number	joint: interaction $F_{(36,2106)} = 2$, $p = 0.03$; outcome: block length $F_{(2,117)} = 5$, $p = 0.01$, interaction $F_{(36,2106)} = 4$, $p < 0.001$	post-hoc t-tests with BHF	block length $p > 0.05$ for all
S11E bottom	Mixed ANOVA for block length and block number	joint: interaction $F_{(36,2106)} = 2$, $p < 0.001$; outcome: block length $F_{(2,117)} = 4$, $p = 0.02$, interaction $F_{(36,2106)} = 3$, $p < 0.001$	post-hoc t-tests with BHF	block length $p > 0.05$ for all
S12B	Mixed ANOVA for block length and distance	joint: block length $F_{(8,351)} = 4$, $p < 0.001$	post-hoc t-tests with BHF	block length $p > 0.05$ for all

Continued on next page

Table 1: Statistical results (Continued)

S12C	Mixed ANOVA for block length and distance	joint: block length $F_{(8,351)} = 4$, $p < 0.001$; outcome: block length $F_{(7,307)} = 3$, $p = 0.003$	post-hoc t-tests with BHF	block length $p > 0.05$ for all
S12D	ANOVA for block length	outcome: block length $F_{(8,63)} = 15$, $p < 0.001$	post-hoc Tukey	Outcome: block length $p < 0.001$ for 1-others
S12E top	Mixed ANOVA for block length and switch number	outcome: block length $F_{(2,112)} = 7$, $p = 0.002$, interaction $F_{(36,2016)} = 3$, $p < 0.001$	post-hoc t-tests with BHF	block length $p > 0.05$ for all except 5-50
S12E bottom	Mixed ANOVA for block length and block number	outcome: block length $F_{(2,112)} = 4$, $p = 0.005$, interaction $F_{(36,2016)} = 3$, $p < 0.001$	post-hoc t-tests with BHF	block length $p > 0.05$ for all
S13B	Mixed ANOVA for probability and number distractors	joint: probability $F_{(6,268)} = 3$, $p = 0.006$	post-hoc t-tests with BHF	probability $p > 0.05$ for all
S13C	Mixed ANOVA for probability and number distractors	joint: probability $F_{(6,268)} = 3$, $p = 0.02$	post-hoc t-tests with BHF	probability $p > 0.05$ for all
S13D	ANOVA for probability	outcome: probability $F_{(6,49)} = 3$, $p = 0.007$	post-hoc Tukey	outcome: $p < 0.05$ probability: 30-0,4

Continued on next page

Table 1: Statistical results (Continued)

S13E top	Mixed ANOVA for probability and switch number	$p > 0.05$ for all		
S13E bottom	Mixed ANOVA for probability and block number	joint: probability $F_{(2,112)} = 3$, $p = 0.04$	post-hoc t-tests with BHF	probability $p > 0.05$ for all
S14B	Mixed ANOVA for probability and distance	$p > 0.05$ for all		
S14C	Mixed ANOVA for probability and distance	outcome: probability $F_{(5,124)} = 3$, $p = 0.02$; interaction $F_{(35,868)} = 2$, $p = 0.01$	post-hoc t-tests with BHF	probability $p > 0.05$ for all
S14D	ANOVA for probability	outcome: probability $F_{(6,49)} = 6$, $p < 0.001$	post-hoc Tukey	outcome: $p < 0.05$ probability: 30-0 and 40-0,4,10
S14E top	Mixed ANOVA for probability and switch number	outcome: interaction $F_{(36,1476)} = 2$, $p = 0.003$	post-hoc t-tests with BHF	$p > 0.05$ for all
S14E bottom	Mixed ANOVA for probability and block number	outcome: probability $F_{(2,82)} = 3$, $p = 0.04$; interaction $F_{(36,1476)} = 3$, $p < 0.001$	post-hoc t-tests with BHF	probability $p > 0.05$ for all

Continued on next page

Table 1: Statistical results (Continued)

S15 left	Threshold 95% upper confidence interval	0.16		
	Linear regression	Crossing threshold feature block: distractors = 12.4; Crossing threshold feature random: distractors = 12.9		
	Linear mixed model algorithm*number distractor*trial type	z(80,1280), p¡0.05 for all factors and interactions	post-hoc estimation of marginal means for across factor comparisons	$p < 0.05$ for all algorithm, algorithm*trial type, and algorithm*number distractors
S15 right	Threshold 95% upper confidence interval	0.16		
	Linear regression	Crossing threshold feature block: distance = 12.0; Crossing threshold feature random: distance = 12.6		
	Linear mixed model algorithm*distance*trial type	z(80,1280), p¡0.05 for all factors and interactions	post-hoc estimation of marginal means for across factor comparisons	$p < 0.05$ for all algorithm, algorithm*trial type, and algorithm*distance
