# Supplementary material for: Contextual inference through flexible integration of environmental features and behavioural outcomes
Source: PLoS Comput Biol. 2026 Mar 20;22(3):e1014093. doi: 10.1371/journal.pcbi.1014093 (PMC13029755; doi:10.1371/journal.pcbi.1014093)
Supplement: Attachment — Submitted filename: Passlack_response_to_reviewers.pdf [file pcbi.1014093.s018.pdf]

Reviewer's Responses to Questions

We thank the Editor and all reviewers for their careful and constructive feedback. We believe that addressing these comments has substantially improved the clarity, scope, and biological grounding of the manuscript.

In particular, the revisions have clarified the conditions under which contextual inference is required, the algorithmic interaction between feature-based and outcome-based inference during learning, and the relationship of the proposed framework to existing experimental and computational work. We have also added new analyses and discussion to make the model's mechanisms, limitations, and experimental implications more explicit.

We hope that the revised manuscript satisfactorily addresses the reviewers' concerns, and we are grateful for the opportunity to revise and resubmit our work. Below, we provide a point-by-point response to each of the reviewers' comments. The original comments are shown in **black**, and our responses are shown in **blue** for clarity. Where informative, we also indicate the relevant page and line numbers in the revised manuscript.

Comments to the Authors:

Reviewer #1: Summary:

This work presents a model of contextual inference which combines feature-based and outcome-based inference. The authors demonstrate that this joint model succeeds in inferring context even in low SNR situations, while the feature-based inference model alone fails as the contexts become more similar. They connect these two distinct forms of inference to the hippocampus and prefrontal cortex, and demonstrate that inactivating the outcome-based inference inhibits the formation of hippocampal "splitter" cells, just as inactivating PFC inhibits their formation experimentally. The work provides a novel mechanistic framework for understanding PFC-hippocampal interactions during context-dependent learning. This is an interesting model and story. However, there are some additional considerations about when and why this joint model provides a benefit that are required.

We thank the reviewer for their thoughtful and constructive evaluation of our work, in particular the detailed focus on when and why joint inference provides a benefit, and on the importance of clarifying the dependence of learning on trial structure.

In revising the manuscript, we have addressed these concerns by adding new analyses that explicitly examine the role of block length, trial stochasticity, and probabilistic trial structure, and by clarifying how outcome-based and feature-based inference interact during learning. We have also revised the text and figures to improve clarity and accessibility throughout.

Below, we respond to each point in turn and indicate where the manuscript has been revised to reflect these changes.

Major Points:

1. I would like to see the authors examine in more detail the dependence of learning on the "blocked" trial structure. I think it would be valuable to see the model's learning performance for random trials, interleaved trials, and blocked with varying levels of added stochasticity. Critically, the lack of these tests makes it a bit vague as to what exactly is necessary for outcome inference to have a benefit. Further, since naturalistic behaviors are not so strictly blocked, and the authors propose that HPC and PFC act as feature and outcome inference, then it should generalize to non-blocked (or at least less strictly blocked) trial structures. This is my biggest sticking point. I would like to see some sort of investigation into this and more discussion about how important blocking is for outcome inference. It doesn't need to be a sweep over all sorts of parameters in the trial structure, but I would like to see the failure in a couple common non-blocked trial structures, and also see how it approaches that failure (is it sudden or gradual?).

We agree that clarifying the dependence of learning on trial structure is essential. We have therefore added a dedicated analysis examining the performance of feature inference (FI), outcome inference (OI), and joint inference across a range of training regimens that progressively relax strict blocking (Results, p.19-20, lines 732-757, Fig S11-S14).

Specifically, we now evaluate performance for different block lengths, including blocks as short as a single trial (Results, p.19-20., lines 732-757, Fig S11-S12), as well as probabilistic trial structures in which each block contains a mixture of trial types (Results, p.19-20, lines 732-757, Fig S13-S14).

These analyses show that the benefit of joint inference is robust to both block length and trial-type probability, and does not depend on the presence of long, strictly homogeneous blocks. Importantly, this robustness arises from the structure of the task itself. In the cued T-maze, each trial permits multiple attempts within a trial ("correction trials"), allowing outcome inference to acquire an accurate outcome map from a single reward experience. As a result, even when block length approaches one trial or trial identity becomes highly probabilistic, outcome inference remains more stable than feature inference alone, enabling the joint algorithm to reduce the number of attempts required at the first reversal. At the same time, these analyses reveal a critical nuance: as trial structure becomes maximally inconsistent (block length ≈ 1 or trial-type probability ≈ 50%), the ability of OI alone to reliably solve the task degrades substantially, reflected in a sharp reduction in the fraction of OI agents that complete the task within the allotted runtime.

In contrast, joint inference remains stable under these conditions, indicating that having two partially independent sources of contextual evidence can stabilise learning even when neither source is individually reliable.

We now discuss this explicitly in the manuscript, clarifying that blocking per se is not required for joint inference to provide a benefit. Rather, blocking serves as a proxy for temporal consistency in latent context, which allows outcome information to stabilise context estimates during learning (Discussion, p.25-26, lines 997-1018).

We further note that such temporal consistency is common in naturalistic settings, where environmental contingencies typically persist over short timescales even when trial identity is not strictly blocked.

When this consistency is reduced, outcome inference alone becomes unreliable, but interaction with feature inference allows joint inference to continue supporting learning.

2. Paper was at times difficult to follow, I'll include specific details in my minor points section. Figures could also use some cleaning up, there is text of different sizes in some places, lines are a bit thin and hard to read. No units in colorbar of Figure 6A. Also, should use a single continuous colormap for 6A as opposed to a tricolor one, as firing rate is a continuous variable and the transition areas of the current colormap (.4 and .8) are arbitrary. .3 vs .5 should look like the same difference as 0 to 0.1.

We thank the reviewer for highlighting these clarity and presentation issues. We have revised both the manuscript text and the figures to improve readability and consistency throughout.

First, we have edited the manuscript for clarity and flow, including tightening phrasing, adding signposting sentences, and clarifying model terminology and transitions between sections. These changes are reflected throughout the Abstract, Results, and Discussion sections.

Second, we have updated all figures to ensure consistent font sizes, line weights, and formatting, and to meet journal guidelines for minimum text size. We have also added units to all colour bars, including Figure 7A (previously Figure 6A), and clarified figure captions where definitions or interpretations were previously ambiguous.

Regarding the colormap in Figure 7A (previously Figure 6A), we retained the original colormap to facilitate comparison with prior experimental and modelling studies of hippocampal place and splitter cell activity that use a similar visual convention. To address the reviewer's concern directly, we now include an additional version of this figure using a continuous colormap in the Supplementary

Material, demonstrating that the reported effects do not depend on the specific choice of colormap (Fig S16).

Together, these changes substantially improve the readability of both the text and figures, while preserving comparability with existing literature.

3. I think it would be important to show performance curves during the training stage, again for different trial structures as in point 1.

We agree that visualising learning dynamics during training is important. We have therefore added performance curves showing agent behaviour across training for the block training structure (Results, p.16-17, lines 598-612, Fig S3-S6) and different trial structures (Results, p.19-20, lines 733-757, Fig S11-14D,E).

Specifically, we now plot the number of attempts required across trials during learning, highlighting how performance evolves within and across blocks and around the first trial-type switch (Results, p.16-17, lines 598-612, Fig S3-S6). These curves show that joint inference selectively rescues performance at the first trial-type switch, leading to sustained improvements both within blocks and across subsequent switches. This improvement corresponds to stabilisation of feature maps for the initially encountered trial type during early reversals, as illustrated in Figure 6.

As shown in our analysis of trial-structure dependence, these learning dynamics are qualitatively unchanged across block lengths, such that plotting separate learning curves for each block length does not reveal additional structure beyond what is already captured by the block-independence of performance.

Together, these additions make explicit how joint inference alters learning dynamics over time, rather than only improving asymptotic performance.

4. The results for Figure 5-S1B and Figure 5-S1C are strange to me. How would adding a reward (in a different location) increase environmental overlap? Further, if the agent is forced into the correct arm of the maze, the SR for each context must be different, so why didn't this improve performance of the FI algorithm? I think these need a bit more investigation and explanation.

We agree that these results require careful explanation, and we have revised both the figures (now Figure S10B,C) and the accompanying text to clarify the underlying mechanisms.

First, we now explain explicitly why adding a reward feature can increase effective environmental overlap. When reward is included as a feature, the ideal successor representation for each context contains identical reward predictions along the shared stem of the maze, such that reward expectancy becomes an additional shared feature alongside spatial location. As a result, rather than separating contextual representations, reward features can increase overlap between the two maps in regions where trajectories are shared. This mechanism is now described explicitly in the Results (Results, p.19, line 717-722) and illustrated schematically in Figure S10B.

Second, when the agent is forced into the correct arm, we clarify why this does not necessarily improve feature inference performance. Under these conditions, the agent continues to attempt to reach the previously rewarded location despite the presence of a barrier, and must therefore update its transition model rather than simply unlearn reward location. Because the feature inference algorithm updates its representations based on predicted transitions and occupancy, this constraint prevents a clear divergence of feature-based maps between contexts, even though the optimal trajectories differ. We have expanded the explanation of this effect in the Results (Results, p.19, lines 722-729) and added schematic illustrations to make this distinction clear (Fig S10C).

Together, these additions clarify that the observed effects arise from how reward expectations and transition structure interact with successor-based representations, rather than from a failure of the algorithms to distinguish contexts per se.

5. This work should be better contextualised. The discussion/introduction should be extended to include discussion on working memory/contextual studies of hippocampal function, as these appear directly relevant. A few examples: I. Lee and R. P. Kesner. Nature neuroscience, 2002. ; Lee and Kesner. Behavioral neuroscience, 2003.; Gilbert and Kesner. Behavioural brain research, 2006.

There is also recent RL contextual modelling work of hippocampal function that appears related to the present work and thus relevant to discuss and contrast (Pedamonti et al. BioRxiv: Hippocampus facilitates reinforcement learning under partial observability).

We agree that broader contextualisation within the hippocampal working memory and contextual learning literature is important. We have therefore expanded both the Introduction and Discussion to situate our framework more explicitly within existing experimental and computational work.

In particular, we now introduce specifically how our work fits into existing literature on contextual inference, specifying what makes a task a contextual inference task, within the introduction and methods section (Introduction, p.2, lines 9-17; Methods p.3-4, lines 93-101, Figure 1). Within the discussion section, we further discuss classic studies demonstrating hippocampal involvement in contextual processing and working memory demands in maze-based tasks (e.g. Lee and Kesner, 2002; Lee and Kesner, 2003; Gilbert and Kesner, 2006), and relate these findings to the requirement for maintaining contextual information when cues are partially observable, highlighting the conditions under which hippocampal involvement is predicted in our framework (Discussion, p.26, lines 1019-1092).

We have also added discussion of recent reinforcement-learning-based models of hippocampal function under partial observability, including work by Pedamonti et al., and contrast these approaches with our framework. Specifically, we have added relevant references including work by Pedamonti et al., to the introduction (Introduction, p.2, lines 23-39 and line 45). Additionally, we clarify how our model complements existing latent-state and recurrent-network approaches by providing a mechanistic account of how outcome-based signals can stabilise feature-based contextual representations during learning, rather than replacing prior models (Discussion, p.28-29, lines 1136-1205).

Together, these revisions more clearly position the present work within the broader literature on hippocampal working memory, contextual inference, and reinforcement learning, and clarify how the proposed framework complements rather than duplicates prior approaches.

Minor Points:

1. From a writing perspective, the flow can be a bit choppy. I will give a couple of examples, but it exists throughout the text. In the abstract, “the ability to use the context we are in to flexibly adjust our decision-making is vital for navigating a complex world” probably reads better simply as “the ability to use context to flexibly adjust decision-making is vital for navigating a complex world”. Later in the abstract, “supporting feature-based….” Should probably be “we demonstrate that supporting…” or “we show that supporting…”. In general I think some more brevity and directness would benefit the manuscript.

We agree, and we have revised the manuscript to improve brevity, flow, and directness throughout. In particular, we have edited the Abstract to address the specific examples raised by the reviewer and have performed an additional round of proofreading to tighten phrasing and improve readability across the main text.

2. At times it is unclear exactly what version of the model is being discussed, for example, I was confused if OI is disabled for the final paragraph of page 11

We thank the reviewer for highlighting this ambiguity. We have clarified throughout the Results and Methods which components of the model are active in each analysis, and have made explicit when outcome inference is disabled versus when joint inference is used (example previously page 11 final

paragraph – now Results, p.19, lines 704-707). This clarification is also now stated explicitly at the relevant points in the Results section.

3. When referring to “behavioral performance”, it is not always clear what metric you are referring to. …”had a marked behavioral effect – it resulted in a decrease in the inferred probability of the context following the cue on correct trials” is not a behavioral measure. How exactly does this inability to contextualize translate to the actions of the agent, and how is that difference quantified?

We agree and have revised the wording to avoid conflating inferred probabilities with behavioural measures. We now refer explicitly to effects on contextual inference, and quantify behavioural performance in terms of measurable agent behaviour, such as the number of attempts required to reach reward (example Results, p.19, lines 697-700). These changes have also been made throughout the Results section where performance is discussed.

4. What happens when OI is left on (after the first 200 trials)?

We now address this explicitly. Leaving outcome inference active beyond the learning phase does not improve performance, and when applied during random trial structures can impair performance. We have added a supplementary analysis illustrating this effect and describe it in the Results (Results, p.17, lines 613-632, Fig S7).

5. What does “difference in splitter probabilities” mean in Figure 6C? Is this just the fraction of splitters?

We have clarified this definition in the text accompanying Figure 7C (previously 6C) (Results, p.22, lines 843-845) and with an additional schematic in Fig 7D. “Difference in splitter probabilities” refers to the difference in posterior probability used to weight the activity of each cell in its preferred versus non-preferred context, rather than the fraction of splitter units. This metric is closely related to the splitter-cell measure used elsewhere in the manuscript, but isolates context-dependent modulation of activity without incorporating future occupancy predictions. The definition is now stated explicitly in the text accompanying Figure 7C.

Reviewer #2: Summary
Passlack and MacAskill's study explores how the brain performs contextual inference - the ability to distinguish between hidden contexts and adjust behavior accordingly. The authors focus on two computational strategies: a feature inference (FI) model, which relies on environmental cues, and an outcome inference (OI) model, which relies on behavioral outcomes, and test the performance of these models in differentiating contexts during learning.

The authors propose a joint inference model that combines both strategies during learning. This hybrid approach allows agents to form distinct contextual representations and improves performance in tasks where contextual signals are weak or noisy. The model is tested on several behavioral paradigms, including a cued T-maze, delayed non-match to sample, and structured paired-associates tasks. In each case, the joint model outperforms individual strategies, especially when contexts are similar or overlapping.

The study draws comparisons with neuroscience findings: the joint model reproduces patterns of hippocampal activity known as splitter cells, which fire differently depending on context. Removing outcome inference during learning mimics (somewhat) the loss of these cells observed in experiments where prefrontal input to the hippocampus is disrupted. The authors conclude that outcome-based signals from the prefrontal cortex may support the hippocampus in forming stable, context-specific representations.

Overall, the manuscript is well written and clearly visualized. The model offers a novel computational framework that explains how the brain might integrate environmental features and behavioral outcomes to perform flexible, context-dependent learning. It largely aligns with experimental data and provides insights into the neural mechanisms underlying contextual inference.

We thank the reviewer for their careful reading of the manuscript and for their constructive comments. In response, we have revised the Introduction, Results, and Discussion to clarify why the task requires inference under partial observability despite the presence of an explicit cue, and to refine the conditions under which hippocampal and prefrontal involvement is predicted in our framework. We have also expanded discussion of relevant experimental findings and clarified terminology where needed. Together we agree that these revisions substantially improve the manuscript.

We address each comment below and indicate where the manuscript has been revised accordingly.

Major comments

1. The manuscript aims to address the problem of contextual inference, which typically involves identifying hidden task states based on behavioral or environmental variables. However, in the T-maze paradigm used here, the context appears to be explicitly signaled to the agent via a visible cue. This raises a concern about whether the task truly requires inference. For example, hippocampal place cells are known to remap in response to sensory changes such as alterations in environmental color (e.g., Anderson and Jeffery, 2003; PMID: 14523083), but this reflects a response to overt sensory input rather than inference of a hidden state. Could the authors clarify how their model captures the process of inferring latent context, given that the agent is provided with an explicit cue that directly indicates the current context?

We thank the reviewer for highlighting the need to clarify why the cued T-maze requires contextual inference. We have expanded both the Introduction, Methods, Results and Discussion to make this point explicit (Introduction, p.2, lines 9-17; Methods, p.3-4, lines 93-101, Figure 1; Results, p.11, lines 345-347; Discussion, p.26-27, lines 1024-1077).

In the task studied here, although contextual identity is indicated by a cue, this cue is not observable at the time point at which the critical decision is made. The cue is encountered earlier along the central stem of the maze and is spatially and temporally separated from the choice point and reward delivery.

As a result, the state at the decision point is partially observable, and correct behaviour requires maintaining and inferring contextual information beyond the duration of the sensory cue. We now

state this explicitly both in the Introduction and in the task description in the Methods and Results (Introduction, p.2, lines 9-17; Methods, p.3-4, lines 93-101, Figure 1; Results, p.11, lines 345-347).

We further clarify that within our framework, hippocampal remapping is interpreted as a consequence of contextual inference rather than a purely sensory response. Specifically, remapping reflects the inference that current observations are inconsistent with expectations derived from previously learned representations, leading to activation of a different contextual map (Discussion, p.26-27, lines 1024-1077). We have added an explicit discussion distinguishing sensory-driven remapping from inference-driven remapping, and relate this interpretation to prior work showing that hippocampal representations can reflect inferred context even when sensory changes are transient or indirect.

Together, these additions clarify that the task requires inference of a latent contextual variable under partial observability, despite the presence of an explicit cue earlier in the trial.

2. In a cued continuous T-maze, Catanese et al. (2012; PMID: 22535656) dissociated splitter cell activity and behavioral choice: when the cue was presented late on the central stem (but still early enough for the animal to make the correct choice) splitter activity continued to reflect the previously learned trajectory until after the decision had been made. A similar absence of splitter activity was reported by Berke et al. (2009; PMID: 19144741) in a cued plus maze. One interpretation of these findings is that cue-guided behavior may not depend on hippocampal processing. If this is the case, it raises questions about the authors' claim that feature inference (FI) depends on the hippocampus and undermines their observation of splitter cell activity. Could the authors comment on how their model accounts for these findings, and whether FI in their framework necessarily reflects hippocampal involvement in cue-driven behavior?

We agree with the reviewer that cue-guided behaviour does not necessarily require hippocampal processing when the cue is fully observable at the time of decision-making. We have revised the Discussion to make this distinction explicit and to clarify the conditions under which hippocampal involvement is predicted in our framework (Discussion, p.26-27, lines 1024-1077).

Within our model, feature inference reflects hippocampal processing specifically in situations where contextual information must be maintained or inferred beyond the availability of the sensory cue. When a cue is fully observable at, or immediately preceding, the decision point, behaviour can be supported by direct cue–response associations without requiring contextual inference, consistent with the findings of Catanese et al. (2012) and Berke et al. (2009). We now discuss these studies explicitly and clarify that our framework does not predict a necessary role for hippocampus in such cases.

At the same time, these studies also demonstrate that hippocampal splitter activity can persist even when it is not required for task performance, and that such activity can depend on inputs from frontal regions. We relate this to our model by noting that outcome inference can drive hippocampal context selectivity during learning even when cue-guided behaviour itself does not depend on hippocampus. We now discuss this correspondence explicitly in the Discussion, including reference to work showing that disruption of prefrontal inputs abolishes hippocampal splitter activity without impairing performance (Discussion, p.28, lines 1106-1135).

Together, these clarifications narrow our claims and show that the proposed framework accommodates both cases: cue-guided tasks that do not require hippocampal inference, and partially observable tasks in which hippocampal involvement is necessary to maintain contextual information across time.

Minor comments

1. Please include line/page numbers for ease of corrections.

We apologise, and agree. We have included line and page numbers in the revised submission, and have referenced our edits using these throughout our reply.

2. Given the discontinuous nature of the T-maze design used in the study, it is not possible to

distinguish between retrospective and prospective splitter cell activity. However, Ito et al. (2015; PMID: 26017312) showed that lesions to the nucleus reuniens specifically abolished prospective splitter signals in CA1. Can the authors comment on whether the reduced splitter activity observed in the FI model, relative to the joint model, reflects a retrospective or prospective signal, or whether this distinction can be inferred or speculated upon within their framework?

Despite the discontinuous nature of the T-maze used here, where the agents being teleported from the end location of the maze to the start of the maze, it is still possible to investigate whether activity is driven by retrospective information coming from the past choice or prospective information predicting the future choice. As a result of agents being teleported to a pre-cue location at the start of the maze, inference in the start location of the maze is entirely retrospective. Following presentation of the cue the retrospective component becomes mixed with the prospective component. However, importantly, in our simulated random-configuration condition there is no stable trial-to-trial contextual variable that could support an Ito-style retrospective splitter signal, because the previous trial's trajectory/cue identity does not predict the current trial's trajectory. Within our framework, stem "splitter-like" activity therefore arises from within-trial latent-state inference conditioned on the current configuration, and from the resulting conditional predictions over future states and outcomes (a successor-style, forward-looking representation). On this interpretation, the reduced splitting under the FI model relative to the joint model reflects less precise state inference and hence less divergent conditional predictions, rather than a shift from prospective to retrospective coding. We have clarified this limitation and interpretation in the Results (Results, p.21, lines 797-811).

3. It is well established that hippocampal place cells can remap, and animals can respond to contextual changes, even in the absence of a structured task, for example, during free foraging (Anderson and Jeffery, 2003; PMID: 14523083). Does the author's model require the presence of task structure to function, or could it also account for more general forms of remapping?

We agree that hippocampal remapping can occur in the absence of an explicit task. We have clarified this point in the Discussion by noting that, in our framework, feature inference does not depend on task structure per se. Rather, remapping arises whenever current observations deviate sufficiently from the predictions encoded in an existing map, independent of whether those observations are behaviourally relevant. This is now highlighted in the discussion (Discussion, p.27, lines 1085-1092).

We now explicitly state that the tasks studied in the manuscript are used because they allow behavioural readouts and comparison to experimental paradigms, but that the underlying feature inference mechanism can, in principle, support remapping during unstructured exploration. We further clarify that outcome inference serves to stabilise remapping when it is behaviourally relevant, rather than to generate remapping in response to sensory features itself.

4. Can the authors offer specific experimental predictions based on their model? For instance, in a task designed to dissociate FI and OI contributions, inactivation of the prefrontal cortex selectively impair performance on components relying on outcome inference? Specific, testable predictions would greatly strengthen the value of the model to the experimental community.

We thank the reviewer for this suggestion and have added a dedicated section outlining experimentally testable predictions. In this section, we describe how the model predicts differential effects of disrupting prefrontal input depending on whether contextual information is partially observable and whether disruption occurs during learning or after representations have been established (Discussion, p.31, line 1254-1294).

5. "Therefore in this instance, past outcome is not informative for behaviour, and so we reasoned that OI agents would not be ale to perform this version of the task." Typo in 'able'.

We have corrected this error in the revised manuscript.

Reviewer #3: General assessment
This manuscript proposes a computational framework combining a feature inference (FI) and an outcome inference (OI) module to account for context-dependent learning in a cued T-maze task. The model aims to capture hippocampal (FI) and prefrontal (OI) contributions to contextual inference. While technically competent and clearly written, the biological motivation and interpretability remain limited. The behavioral paradigm is not clearly tied to a hippocampal-dependent task, and the computational novelty relative to existing latent-state or cue-based inference models is modest. Overall, the work presents an interesting algorithmic idea—joint inference of context from features and outcomes - but it does not convincingly demonstrate what we learn about hippocampus, prefrontal cortex, or specific behavioral phenomena. Strengthening the biological rationale, clarifying the model's mechanisms, and situating it more explicitly within existing literature on cue-based learning, contextual inference, and hippocampal representations would substantially improve the manuscript.

We thank the reviewer for the careful and detailed critique. We agree that the biological framing and interpretability need to be explicit and carefully bounded. In revising the manuscript, we have focused on clarifying (i) the conditions under which our mapping of FI to hippocampal processing is intended, (ii) the algorithmic mechanism by which outcome-based inference supports feature-map learning, and (iii) how the framework relates to existing latent-state and cue-based contextual inference models. We have also revised phrasing throughout to avoid overstating novelty or biological specificity.

1. Major comments
The link between the proposed modules and hippocampal–prefrontal circuitry is tenuous.
The involvement of the hippocampus in the task modeled—a 2-arm maze where an early cue indicates which arm is rewarded - needs to be specified (e.g. Kim, Lee & Lee, 2012; Ainge et al., 2007). Many radial- and T-maze behaviors of this type rely more on anterior cingulate or medial prefrontal regions (Seamans, Floresco & Phillips, 1995). Consequently, the mapping of FI → hippocampus and OI → PFC requires some unpacking.
If Kim et al 2012 is taken as the closest empirical precedent, hippocampus is required only when the cue disappears before the choice, implying a memory or contextual maintenance process. In contrast, the model updates its inferred context continuously at each state and makes the sole decision at the final junction, assuming ongoing re-inference that lacks a clear biological correlate. These assumptions—decision timing and continuous context updating—should be acknowledged explicitly, as they constrain the biological validity of the framework.
More generally, the paper does not clearly show what new insight it provides about hippocampal or prefrontal function. The proposed combination of successor-representation-like inference and outcome-based contextual labeling reads more as a generic algorithmic proposal than a testable neural hypothesis.

We are grateful to the reviewer for this careful and detailed critique. We agree that the biological interpretation of the framework needs to be stated more narrowly and explicitly, and that our original presentation did not sufficiently separate algorithmic abstraction from biological claims. In revising the manuscript, we have therefore focused on clarifying the scope and limits of the hippocampal–prefrontal mapping, the modelling assumptions involved, and the specific biological insights the framework is intended to provide.

(i) Relationship between model components and hippocampal–prefrontal circuitry

We agree that the mapping of feature inference (FI) to hippocampal processing and outcome inference (OI) to prefrontal processing should not be interpreted as universal or exclusive. In the revised manuscript, we now explicitly restrict this interpretation to tasks in which contextual information is partially observable at the time of choice and must be maintained or inferred beyond the availability of sensory cues (Discussion, p.27-28, line 1021-1135).

In particular we have expanded the Discussion to clarify that cue-guided behaviour does not necessarily depend on hippocampus when cues are fully observable at the decision point, consistent with prior work in cued T-maze and plus-maze paradigms. Instead, hippocampal involvement is predicted specifically in situations requiring contextual maintenance or inference across space or time. This framing is now made explicit in the sections discussing hippocampal and prefrontal

contributions to contextual inference, and the cued T-maze task is positioned accordingly within this literature.

(ii) Modelling assumptions and abstraction

We agree that the modelling assumptions regarding decision timing and context updating constrain the biological interpretation of the framework and should be acknowledged explicitly. In the revised manuscript, we now clarify that inference is updated at the level of state transitions in a discretised environment, and that this is a computational abstraction introduced for tractability rather than a literal description of continuous neural dynamics or decision processes.

We have added a dedicated discussion of these assumptions in the Limitations section (Discussion, p.30, line 1206), where we explicitly outline how discretisation of space, inference updates across states, and simplified decision timing limit the biological specificity of the model. These clarifications are intended to make clear which aspects of the framework are mechanistic proposals and which are modelling conveniences.

(iii) Testable neural hypotheses and contribution

We agree that the manuscript should more clearly articulate what new, testable insight it provides about hippocampal and prefrontal function. To address this, we have added a dedicated section outlining experimentally testable predictions (Discussion, p.31, line 1254). In this section, we describe how the framework predicts differential effects of disrupting prefrontal input to hippocampus depending on whether contextual information is partially observable and on whether disruption occurs during learning versus after representations have been established.

These predictions are framed in terms of experimentally accessible manipulations, including cue–choice distance, presence of distractors, and temporally specific perturbations of prefrontal or thalamic inputs. We hope this clarifies that the goal of the framework is not to propose a generic algorithm, but to generate falsifiable hypotheses about hippocampal–prefrontal interactions during contextual learning.

2. Model exposition and transparency
The presentation could be streamlined. Clarifying the mechanism under study would improve readability and help the reader focus on the key computational idea.
• Explain clearly how OI and FI are combined for decision-making: both modules infer a context posterior, these are averaged during early trials, and the resulting posterior selects the context-specific TD map for action selection.
• Explicitly define the feature vector used by FI (positions + cue channels, with cue over-representation) and the state representation used by OI (tabular positions).
• Include the essential inference and TD equations in the Results section rather than only in Methods.

We thank the reviewer for this comment and agree that the model description in the original manuscript did not sufficiently foreground the core computational mechanism. We have therefore revised the Results and Methods to make the interaction between feature inference (FI) and outcome inference (OI) clearer and more self-contained.

First, we now explain explicitly how FI and OI each infer a posterior over context, how these posteriors are combined during early learning, and how the resulting joint posterior selects the context-specific value representation used for action selection. This description is now included directly in the Results section alongside the behavioural analyses (Results, p.11-12, lines 388-416 and p.15-16, lines 548-560), rather than being confined to the Methods, so that the mechanism is clear at the point where its behavioural consequences are presented.

Second, we now define the representations used by each module explicitly. In particular, we clarify that FI operates over a feature vector comprising spatial location and cue channels (with explicit over-representation of the cue), whereas OI operates over a tabular representation of spatial state. These definitions are now stated consistently in the Results (Results, p.11, line 352-354 and 381-385), and

we have edited the surrounding text to avoid ambiguity about which representation is being discussed.

Finally, we agree that accessibility is improved when the essential inference and temporal-difference learning equations are presented where the algorithms are introduced. We have therefore moved the key equations into the Results section (Results, p.11-12, lines 388-416), while retaining implementation details and parameter settings in the Methods. This ensures that the Results provide a self-contained account of the model logic without requiring extensive cross-referencing.

3. Biological grounding of the task
The choice of task needs stronger justification. Several studies have demonstrated cue → response T-maze learning without block pre-training (Barnes et al., 2011; Kim et al., 2012; Arlt et al., 2022). The manuscript currently over-states the absence of models for “context-dependent learning without externally provided context signals.”
Here, “context” corresponds to which side of the maze is rewarded - essentially a cued two-alternative-choice situation. The authors should situate their work within the extensive literature on cue-based decisions, latent-state inference (e.g. IBL), and classic context-learning models in spatial navigation (e.g. Tolman’s hidden-state frameworks).

We thank the reviewer for this critique and agree that the original manuscript did not sufficiently distinguish between cue–response tasks and partially observable contextual inference tasks, nor did it adequately situate the cued T-maze within the broader literature on cue-guided choice and latent-state inference.

In the revised manuscript, we now clarify more explicitly why the cued T-maze studied here constitutes a partially observable contextual inference problem. Although a cue is present, it is encountered earlier in the trial and is not observable at the time of the critical decision at the choice point, nor at reward delivery. As a result, correct behaviour requires maintaining or inferring contextual information beyond the sensory cue. We now state this explicitly in the task description (Methods, p.3-4, lines 82-101, Fig 1) and Introduction (Introduction, p.2, lines 9-17), and we discuss how this differs from variants in which cues remain visible at choice and behaviour can be supported by direct cue–response associations (Discussion, p.26-27, lines 1024-1077).

We also agree that our original wording overstated the absence of models capable of context-dependent learning without externally provided context signals. We have therefore softened this claim and revised the Introduction and Discussion to explicitly acknowledge existing latent-state and block-prior inference frameworks, including IBL-style approaches and Bayesian accounts discussed in Gershman’s work. In the revised text, we clarify that our contribution is not the existence of contextual inference per se, but a mechanistic account of how feature-based and outcome-based inference can interact during learning to stabilise distinct contextual representations when contextual signals are weak or overlapping, particularly in multi-action spatial and sequential tasks (Discussion, p.28-29, lines 1136-1205).

Together, these changes are intended to place the task and the model within the appropriate existing literature, and to more carefully delimit the scope of our claims.

4. Clarify the OI module’s role
The contribution of the OI component is described inconsistently. In the model, the OI posterior is averaged with the FI posterior during the first 200 trials; there is no explicit dynamic stabilization signal from OI to FI. It should be stated clearly that the joint posterior provides cleaner early context labels, allowing FI to learn distinct maps, after which FI operates independently.
The biological analogy (PFC providing a stabilizing signal to HPC) could then be discussed as a supervised labeling phase. The authors should also explain why OI input is required continuously in later tasks but only during learning in the T-maze.

We thank the reviewer for highlighting that the role of the outcome inference (OI) module was described inconsistently. We agree that this required clearer exposition, and we have revised the manuscript to state the mechanism more explicitly and consistently.

In the revised manuscript, we now state directly that FI and OI each infer a posterior over context, and that during early learning these posteriors are combined to form a joint context estimate that selects which context-specific value representation is used for action selection. This combined estimate provides more reliable early context assignments, allowing FI to learn distinct feature-based maps (Results, p.15-16, lines 545-569 and p.20, lines 780-787).

We also clarify that in the cued T-maze setting OI is not intended to provide a continuous stabilising signal throughout later performance. Rather, its key role is during learning, when feature-based representations are most vulnerable to being corrupted at early reversals. Once stable feature-based maps have been learned, FI can support performance independently in this task. This learning-versus-performance distinction is now stated explicitly in the Results where joint inference is introduced (Results, p.15-16, lines 545-569 and p.16-17, lines 598-626).

We agree that the biological analogy should be framed carefully. In the revised Discussion, we describe OI as providing a stabilising influence on early learning by improving context assignment when cues are partially observable. This can be viewed as analogous to a supervised labelling phase in the limited sense that early context estimates become more reliable, but we do not intend to imply explicit labels or ground-truth context identity. Instead, both FI and OI contribute probabilistically to a joint estimate, and it is this joint estimate that supports reliable learning of feature-based maps (Discussion, p.25, lines 967-996).

Finally, we clarify why ongoing outcome-based support is required in some of the other tasks examined but is not required beyond learning in the cued T-maze. In the revised text, we explain that tasks with more contexts and/or greater overlap can require persistent outcome-based information to support reliable context assignment, whereas in the two-context cued T-maze, once feature-based maps are established, FI alone is sufficient for performance (Results, p.23, lines 906-914).

5. Model evaluation and interpretation
• “Training” vs “testing.” These terms are potentially misleading since the “testing” phase still includes reward feedback; only the trial structure changes (random vs blocked). Clarify terminology for consistency.
• “Predictive strategy.” This phrasing is ambiguous. FI performs cue → response mapping within a trial, not across trials. Clarify that “predictive” refers to within-trial cue-based prediction, not cross-trial latent inference.
• Quantitative analysis. It would help to show the relative contributions of FI and OI to the joint posterior and how each fails when used alone. Visualizing the separate context maps MzMz and CzCz would make this clearer.

We thank the reviewer for these comments and agree that clearer terminology and more explicit interpretability are important. We have therefore revised the manuscript to address each point directly.

First, we agree that “training” versus “testing” language was misleading because reward feedback is present throughout and the primary manipulation is trial structure (blocked versus random). In the revised manuscript, we now use more precise language that distinguishes between different training regimens and evaluation periods used for quantification, without implying an absence of learning (Examples: Results, p.14, lines 487-488 and Fig 3B legend; Fig 8B,C,E,F).

Second, we agree that our use of the phrase “predictive strategy” could be misinterpreted as cross-trial latent inference. We now state explicitly that FI is a within-trial cue-based predictive mechanism, rather than a cross-trial outcome-based inference process. This distinction is now clarified at the point where FI is introduced and described in the Results (Results, p.11, lines 363-374).

Third, we agree that readers benefit from seeing how FI and OI contribute to the joint posterior and how each fails when used alone. We have therefore expanded the Results and Supplementary Material to visualise the maps (Results, p.14, lines 497-501, Fig 3E) and posteriors learned by FI and OI separately, and to show how the joint estimate relates to each (Results, p.17-18, lines 633-652, Fig S8-S9). These additions are intended to make when FI-only and OI-only fail under partial observability more transparent, and to clarify how the combined estimate stabilises learning at early reversals.

6. Relation to existing algorithms
The reference to Allen et al. (2024) is intriguing but underdeveloped. Without implementing such automatic arbitration, it would still be informative to analyze how discrepancies between FI and OI posteriors evolve and whether weighting by these discrepancies could explain the observed performance improvements.

We thank the reviewer for this suggestion and agree that the reference to arbitration-style ideas would be more informative if we more directly showed how FI and OI posteriors diverge and converge over learning.

In the revised manuscript, we now explicitly visualise FI and OI posterior confidence over time and around reversals, alongside the joint context estimate (Results, p.17-18, lines 633-652, Fig S8-S9). These analyses show that OI tends to provide higher-confidence context estimates early in learning and following reversals (shown as a large decrease in the probability of the past trial identity after a reversal), whereas FI confidence increases once stable feature-based maps have been established. Presenting these confidence traces makes the evolution of FI–OI discrepancies explicit, without introducing an additional arbitration mechanism.

We also expand the Discussion to relate this pattern to recent work on arbitration between predictive and outcome-based systems, including Allen et al. (2024), while clarifying that our focus here is on stabilising early learning under partial observability rather than proposing a full arbitration framework for asymptotic performance (Discussion, p.30, lines 1238-1253).

In addition, we include a complementary analysis examining how relative confidence in feature inference versus outcome inference varies as task difficulty increases (Results, p.20, lines, 758-787, Fig S15). In this analysis, we show that the point in tasks with sufficient distractors or distances, where outcome inference confidence exceeds feature inference confidence, closely coincides with where joint inference yields a marked behavioural improvement relative to feature inference alone.

This correspondence provides further evidence that the benefit of joint inference emerges specifically when feature-based estimates become unreliable, and outcome-based information provides a more stable contextual signal. We emphasise that this analysis is descriptive rather than mechanistic: while it is consistent with the idea that relative confidence could be used to arbitrate between inference strategies, the model does not implement such an arbitration process. Instead, these results help to characterise when and why outcome-based support is beneficial during learning under partial observability.

7. Claims and literature positioning
At several points the text claims that no algorithms can perform context-dependent learning without an externally provided context signal. This is inaccurate: latent-state and block-prior inference frameworks already address this (e.g. IBL; Gershman 2017 Curr Opin Neurobiol). The authors should soften this statement and acknowledge prior work.

We thank the reviewer for pointing out that our original framing overstated the absence of existing models capable of context-dependent learning without externally provided context signals. We agree that this wording was too strong, and we have revised the manuscript accordingly.

In the revised Introduction and Discussion, we now explicitly acknowledge existing latent-state and block-prior inference frameworks, including Bayesian and recurrent-network approaches, and we position our work within this broader literature rather than in opposition to it (Introduction, pg.2, lines 24-28; Discussion, pg.28-29, lines 1136-1205). We also soften the relevant claims throughout to avoid implying uniqueness.

We further clarify that our contribution is not the existence of contextual inference per se, but a specific mechanistic proposal for how outcome-based information can stabilise feature-based representation learning when contextual cues are weak or overlapping, particularly in multi-action

spatial and sequential tasks. This revision is intended to provide a more balanced and accurate literature positioning.

8. “Splitter-cell” analogy
The analogy to hippocampal splitter cells is overstated. Biological splitters encode future trajectory even in cue-free zones (e.g. Ferbinteanu & Shapiro 2003). In contrast, the model’s “splitter-like” activity arises from cue/context features explicitly encoded in the state vector—better described as context selectivity than prospective splitting.
To strengthen this claim:
• Perform position × context decoding or future-turn-conditioned analyses on cue-absent states.
• Clarify how the metric distinguishes “splitter” from “cue” cells.

We thank the reviewer for this careful critique and agree that our original wording risked overstating the correspondence between the modelled activity patterns and experimentally defined splitter cells.

In the revised manuscript, we now qualify the analogy more explicitly and distinguish between context-dependent modulation in the model and the broader set of phenomena often referred to as splitter activity in continuous-maze experiments. We now describe our readout as capturing context-dependent modulation of predictive representations under partial observability, rather than as a complete account of prospective trajectory coding (Discussion, p.28, lines 1109-1135).

We also clarify explicitly in the Results that our splitter analyses focus on locations along the central stem where the cue is no longer present, such that the analysed activity does not reflect instantaneous cue encoding. Instead, context selectivity in these locations arises from maintained contextual estimates that modulate predicted future occupancy. We have revised the surrounding text to make this distinction clear and to avoid conflating cue-related activity with context-dependent predictive activity, as well as clarifying the relationship to prospective splitting (Results, p.21, lines 797-818, Fig 7D).

We believe these revisions make the scope of the analogy clearer while retaining the central point that outcome-based support during learning can promote robust context-dependent modulation in a hippocampal-like predictive representation.

Minor comments
• Define “attempts on block switches.” Specify how this is computed (e.g. number of incorrect trials after a switch until first correct).

We agree that this definition should be explicit. In the revised manuscript, we now define “attempts on block switches” as the number of attempts required after a trial-type switch until the agent first reaches reward under the new contingency. This definition has been added to the Results section where this metric is introduced (Results, pg.11, lines 357-360 and pg.13, lines 453-457).

• Clarify Fig. 2C (y-axis label, caption, and interpretation).

We have revised Figure 3C (previously 2C) to clarify the y-axis label and updated the caption to more clearly explain what is being plotted and how it should be interpreted. The accompanying Results text has also been edited to improve clarity (Results, pg.13, lines 458-463).

• Correct Fig. 5 caption (panel “e” mismatch) and explain how cue representations were computed.

We have corrected the panel labelling in the Figure 6 (previously Fig 5) caption and expanded the Results text to explain how cue representations are computed in the model (Results, pg.18, lines 680-689). This information is now consistent with the Methods descriptions.

• Add statistical notation in figure legends (* $p < 0.05$) and refer to Table 6 for detailed tests.

We have updated the figure legends to include statistical notation where appropriate and now explicitly refer readers to Table S1 for full details of the statistical tests.

• Either justify or remove the speculative “Lévy flight” comment, which is difficult to interpret in a discrete corridor.

We agree that this statement required clarification if kept in. In the revised manuscript, we have removed this statement in order to focus on some of the more important limitations including the assumptions about the environment structure and inference mechanisms (Discussion, p.30, lines 1206-1253).
