# Supplementary material for: Contextual inference through flexible integration of environmental features and behavioural outcomes
Source: PLoS Comput Biol. 2026 Mar 20;22(3):e1014093. doi: 10.1371/journal.pcbi.1014093 (PMC13029755; doi:10.1371/journal.pcbi.1014093)
Supplement: Attachment — Submitted filename: passlack_rebuttal_r2.pdf [file pcbi.1014093.s019.pdf]

Reviewer's Responses to Questions

We thank the Editor and all reviewers for their careful and constructive feedback. We are pleased that two reviewers consider the manuscript acceptable for publication, and we believe that addressing the remaining comments has substantially improved the clarity, scope, and biological grounding of the work.

In response to the additional comments, we have clarified the conditions under which contextual inference is required, the algorithmic interaction between feature-based and outcome-based inference during learning, and the relationship of the proposed framework to existing experimental and computational approaches. We are grateful to the reviewers for their thoughtful suggestions, which have strengthened the manuscript.

We hope that the revised manuscript satisfactorily addresses all remaining concerns, and we appreciate the opportunity to revise and resubmit our work. Below, we provide a point-by-point response to each reviewer comment. The original comments are shown in **black**, and our responses are shown in **blue** for clarity. Where informative, we also indicate the relevant page and line numbers in the revised manuscript.

Comments to the Authors:

Major comments

Biological interpretation and task relevance
(Lines 430, 463–467, 592, 700, 827, 1025, 1059, 1080, 1120–1122)

While the model is internally consistent, the biological interpretation remains challenging. The cued two-arm T-maze used here is not unambiguously hippocampal-dependent, and prior experimental work suggests that reliance on hippocampus versus mPFC/ACC depends strongly on task timing, cue availability, and memory demands (e.g., Kim et al., 2012; Ainge et al., 2007; Seamans et al., 1995). It would strengthen the manuscript to more explicitly delineate which aspects of the modeled behavior are expected to rely on hippocampus versus prefrontal cortex, and under what task constraints.

Relatedly, several claims regarding context-specific representations (e.g., distinct maps, splitter-like activity) would benefit from a clearer distinction between context selectivity driven by explicit cue features and prospective trajectory coding as reported in hippocampal physiology. As currently phrased, some statements may read as broader than what the task and model can directly support.

We agree that hippocampal dependence in cued T-maze variants is contingent on the temporal and spatial relationship between cue availability and the moment of choice, and we have tightened the manuscript to make this boundary condition explicit. In our task, the cue is encountered on the stem but is not available at the choice point, so correct behaviour requires maintaining or inferring contextual identity beyond the duration of the cue. We now frame the hippocampal mapping of the feature inference component in terms of this specific requirement, rather than suggesting that all cued T-maze variants are hippocampal dependent. We further clarify that the outcome inference component is intended as a computational analogue of outcome-driven contextual labelling signals that can be expressed in prefrontal networks, particularly when contextual evidence from sensory features is weak or overlapping. Finally, we have moderated several statements about context-specific representations to distinguish cue-driven selectivity from prospective trajectory coding, and we explicitly state the limits of what can be inferred from this task and modelling abstraction.

In addition to moderating language throughout, we have added an explicit delineation of predicted hippocampal and prefrontal contributions across different task regimes, specifying how cue availability, temporal displacement, and contextual overlap alter the relative contributions of feature-based predictive mapping and outcome-based contextual inference.

Line 1102: “Taken together, the framework suggests a division of labour that depends on task structure. When contextual cues remain available at the decision point and discriminability is high,

behaviour can be supported by direct cue–response policies with minimal reliance on predictive mapping. When cues are temporally displaced from choice or obscured by other features, successful performance requires maintaining or reconstructing contextual state, a regime in which hippocampal-like predictive representations are expected to contribute. Finally, when contextual features are weak or highly overlapping, outcome-based contextual labelling becomes more reliable than feature-based inference, increasing the expected contribution of prefrontal-like outcome signals, particularly during learning or under persistent ambiguity."

Specific requests:

Line 430 (line 435 in revised manuscript): We have revised the introductory framing of the T-maze section to emphasise that this task serves as a baseline regime with relatively high contextual discriminability, rather than as a general proof-of-principle for contextual inference. We also clarified that subsequent manipulations systematically increase contextual ambiguity by reducing cue–choice discriminability.

Line 463 (line 473 in revised manuscript): We agree that the previous wording could be interpreted as implying a general necessity of Bayesian inference for contextual inference. We have revised this sentence to explicitly scope the claim to our modelling framework and set of comparisons.

Line 592 (line 615 in revised manuscript): We have revised this section to clarify that increasing overlap reduces contextual discriminability and to frame the resulting misassignment as a property of the model's predictive representations under ambiguous conditions, rather than as a general claim about contextual inference.

Line 700 (line 716 in revised manuscript): We have revised this sentence to explicitly scope the claim to the modelling framework and to clarify that the impairment refers to reduced separation of context-specific predictive representations under increased ambiguity.

Line 827 (line 847 in revised manuscript): We agree that the biological interpretation should be framed cautiously. We have revised this section to describe the FI–OI interaction as a computational analogue of proposed PFC–HPC interactions and moderated language suggesting necessity. We now emphasise that this mapping is hypothesis-generating and contingent on task structure, rather than a one-to-one circuit model.

Line 1025 (line 1061 in revised manuscript): We have revised this paragraph to more precisely delimit the conditions under which hippocampal involvement has been reported, emphasising temporal displacement of cue information and moderating statements of necessity. This clarifies that our biological mapping is contingent on task structure rather than universal across cued navigation tasks.

Line 1059 (line 1112 in revised manuscript): We have revised this paragraph to avoid implying superiority over simpler paradigms and to clarify that the extensions involve increased structural and temporal demands rather than a qualitatively different form of contextual inference. We also clarify that these differences do not, by themselves, imply uniform hippocampal dependence.

Line 1080 (line 1134 in revised manuscript): We have moderated the wording in this section to describe the feature-based component as exhibiting representational properties that resemble hippocampal phenomena, rather than "capturing" hippocampal representations.

Line 1120 (line 1182 in revised manuscript): We have moderated statements in this section to avoid absolute claims regarding hippocampal necessity and instead describe empirical findings as conditional on specific task variants. This clarifies that our interpretation depends on cue availability and temporal structure rather than on generic properties of T-maze tasks.

Mechanistic clarity of FI–OI interaction
(Lines 430, 649, 700, 722, 827, 939)
The description of OI "supporting" FI would benefit from additional precision. In the current implementation, OI contributes via averaging of posteriors during early learning, rather than via an

ongoing stabilizing or modulatory signal. Explicitly framing this interaction as an early supervised labeling phase -after which FI operates largely autonomously - would help clarify both the computational mechanism and the proposed biological analogy.

We agree that the mechanistic role of OI should be stated more precisely. In the cued T-maze, OI contributes to FI primarily during early learning via joint posterior estimation, which provides more reliable contextual labelling at block switches. This prevents mis-updating of feature-based predictive maps and facilitates their separation. Once context-specific maps are established under high-discriminability conditions, FI alone is sufficient for stable performance and continued OI input does not substantially alter behaviour. We have revised the manuscript to frame this interaction explicitly as a learning-phase contextual labelling mechanism rather than as a continuous stabilising signal.

We further clarify that the persistence of OI contributions in later tasks depends on task structure. In regimes with high contextual overlap or weak feature discriminability, outcome-based inference remains more reliable than feature-based inference, and therefore continues to influence context estimation beyond initial learning. We have added text to make this task-dependence explicit and to delineate the limits of the FI–OI interaction across task regimes.

Specific requests:

Line 545 (559 in new manuscript) and 598 (612 in new manuscript): We have clarified that OI contributes to context assignment only during the initial learning phase via posterior averaging, and that FI subsequently operates independently. We also tightened the description of this mechanism to emphasise that it is limited to shared context estimation rather than direct parameter interaction.

Line 992 (1014 in new manuscript): We have clarified that once context-specific maps are established under high contextual discriminability, FI operates autonomously and that OI contributes primarily during early learning. We explicitly state that the FI–OI interaction is phase- and task-dependent rather than continuously stabilising.

In addition, it would be helpful to explain why OI input appears to be required continuously in later tasks but only during learning in the T-maze, and what this implies about the generality or limits of the proposed framework.

Added at line 1042: We have expanded the Discussion to clarify why outcome-based inference plays only a transient role in the cued T-maze but appears more persistent in the biconditional discrimination task. We now explicitly distinguish between tasks in which feature-based maps become stable and self-sustaining after learning, and those in which context estimates remain more fragile over extended training. In particular, we note that the structured paired-associate task behaves similarly to the T-maze, whereas performance in the biconditional task gradually degrades in the absence of outcome-based recalibration. We present this as an interpretive observation and emphasise that the persistence of OI depends on task structure rather than reflecting a universally required stabilising signal.

Specific requests:

Line 430 (line 435 in revised manuscript): We have revised the introduction of the cued T-maze to clarify that it serves as a high-discriminability baseline regime rather than as a canonical or definitive test of contextual inference. This framing ensures that subsequent discussion of FI–OI interaction is grounded in a controlled starting condition before systematic increases in contextual ambiguity.

Line 649 (line 664 in revised manuscript): We revised this section to clarify that the improvement in first-block-switch performance arises from posterior averaging during early learning rather than from a continuous stabilising interaction. We now explicitly describe how the sharp reduction in OI posterior following unexpected reward omission contributes to a clearer joint context estimate when averaged with FI, thereby reducing misassignment at reversal.

Line 700 (line 716 in revised manuscript): We have revised this sentence to scope the claim explicitly to the modelling framework and to clarify that increased overlap reduces separation of

context-specific predictive maps within FI. We also specify that outcome-based inference mitigates this reduction during early learning, rather than implying a continuous compensatory interaction.

Line 722 (line 739 in revised manuscript): We have clarified that forcing behavioural exploration does not resolve ambiguity in context assignment when posterior separation remains weak, and we have scoped the summary statement to the modelling framework and early learning phase. We now explicitly state that outcome-based integration promotes separation of context-specific predictive maps during early learning, rather than implying a continuous or universally required interaction.

Line 827 (line 849 in revised manuscript): We have further moderated the biological interpretation by describing the FI–OI interaction as a computational analogue of proposed PFC–HPC interactions and by softening language implying direct replication of splitter-cell loss. We now refer to qualitative resemblance in context-map separation rather than to literal modelling of splitter activity, and reiterate that this mapping is hypothesis-generating rather than a circuit-level account.

Line 939 (line 962 in revised manuscript): We have clarified that the improvement observed when integrating OI in this task reflects enhanced stability of context assignment during learning, rather than a continuously required stabilising interaction.

Inference dynamics, decision timing, and trajectory control
(Lines 795, 1025, 1059, 1080)

The manuscript would benefit from greater clarity regarding when decisions are assumed to occur. While the model updates context continuously as the agent progresses through states, behaviorally the task may permit an early commitment upon cue presentation, followed by execution rather than ongoing inference. Clarifying whether decisions are assumed to be made at cue onset, at the junction, or continuously would help align the model with existing decision-making frameworks (e.g., hierarchical or option-based control) and avoid over-interpreting sequential inference in this task.

We have clarified in the Discussion that the model assumes ongoing context inference across states, with actions selected continuously based on the current posterior. We explicitly acknowledge that simpler cue-triggered motor strategies could solve the basic T-maze, and emphasise that our aim is not to model motor commitment per se, but to provide a principled mechanism for maintaining and applying latent contextual information at the point of choice under complete sensory ambiguity. We now make this distinction explicit.

We have also clarified that in the model, context inference selects the active context-specific TD map, which in turn governs action selection at each state. Thus, while contextual evidence is typically acquired at cue onset, the decisive action at the junction reflects the currently active policy rather than an explicit commitment at cue presentation. This makes the hierarchical structure of the model explicit and clarifies the assumed decision timing.

Added at line 1095: "In the present framework, context inference determines which context-specific TD map is active, and this map in turn governs action selection at each state. Although contextual evidence is typically acquired at cue onset, the decisive action at the junction is generated by the currently active policy rather than by a fixed commitment made at cue presentation. In this sense, the model is hierarchical: a latent context estimate selects a policy, and that policy is executed across states unless updated by new evidence."

Specific requests:

Line 795 (line 818 in revised manuscript): We have moderated the description of splitter cells to avoid implying that their activity necessarily reflects ongoing sequential deliberation. We now describe them as prospective in the sense of differing according to upcoming trajectory or behavioural context, without tying this directly to continuous decision updating in the model.

Line 1025 (line 1061 in revised manuscript): We have revised this paragraph to clarify that hippocampal involvement has been reported specifically in task regimes where relevant information is encountered prior to, but not at, the moment of choice. This framing emphasises partial observability

and temporal separation rather than continuous deliberation, and clarifies the assumed decision timing in the model.

Line 1059 (line 1095 in revised manuscript): We have clarified that in the spatial T-maze the decisive action occurs at the junction, and that the sequential structure reflects execution of a context-conditioned policy rather than continuous deliberation. We have also reframed the computational description to emphasise maintenance and application of contextual information across time, rather than ongoing trajectory-level inference.

Line 1080 (line 1134 in revised manuscript): We have moderated the wording to describe the feature-based component as exhibiting representational properties that resemble hippocampal activity, rather than "capturing" hippocampal representations directly. This avoids implying mechanistic equivalence while retaining the intended qualitative comparison.

Comparison to existing models
(Lines 430, 467; Line 1161)
Several statements suggest that the proposed framework addresses limitations of existing contextual inference algorithms. It would strengthen the contribution to more explicitly compare the present approach to latent-state, block-prior, and hierarchical reinforcement-learning models, clarifying what is gained - and what remains limited - by the FI–OI combination relative to this existing literature. Relatedly, statements such as "to confirm that Bayesian inference is necessary…" (Lines 466–467) may read as overly general. It may be more accurate to state that Bayesian inference is necessary within the present modeling framework or set of comparisons, rather than implying necessity for contextual inference more broadly.

We agree with the reviewer and have expanded our discussion of how the present framework relates to existing contextual inference models. In particular, we have added a new paragraph clarifying how the FI–OI framework relates to broader classes of contextual inference models, including Bayesian latent-state and hierarchical reinforcement-learning approaches. We emphasise that our goal is not to propose a fundamentally different inference principle, but to provide a mechanistically transparent decomposition of feature- and outcome-driven contextual signals and to analyse their interaction under varying levels of contextual overlap.

Around at line 1273: "More broadly, our model falls under the category of hierarchical reinforcement learning, which involves grouping multiple state-action pairs together into a single component - often referred to as an option - and then generating a policy based on these higher-level options. Traditionally, hierarchical reinforcement learning models group together multiple state-action pairs that allow for reaching subgoals within the environment to overcome limitations associated with having a large state space, allowing for more efficient initial exploration of the space, as well as faster learning if the structure of the environment or reward location changes [102,103]. Within our implementation, the options can be viewed as the policies defined by each context-dependent TD map (ie turn left vs turn right), however, contrasting with traditional hierarchical models, the separation and use of options is gated through the current inferred latent state. As a result, the hierarchical nature of our model is used for learning and activating distinct policies depending on the context, rather than allowing for better learning within a single context, as is the case in traditional hierarchical reinforcement learning."

Specific requests:

Line 430 (line 435 in revised manuscript): We have revised this sentence to avoid implying that the present framework supersedes existing contextual inference algorithms. The cued T-maze is now described as a baseline task commonly used in studies of contextual inference, rather than as a benchmark against alternative algorithms.

Line 467 (line 477 in revised manuscript): We have revised this sentence to scope the claim explicitly to the present modelling framework and task comparison. We now state that Bayesian inference is necessary within our modelling framework for solving the cued T-maze under the specified manipulations, rather than implying general necessity for contextual inference more broadly.

Line 1161 (line 1230 in revised manuscript): We have removed speculative statements regarding extension to within-context trajectory optimisation and trajectory-level valuation to avoid overgeneralising the scope of the present framework. The Discussion now limits claims to properties directly supported by successor-representation theory and demonstrated within the current modelling analyses.

Quantitative and experimental grounding
(Lines 463–467, 487, 649, 722)
Where quantitative performance metrics are reported (e.g., number of attempts on block switches), comparison to experimental learning rates or behavioral variability would help contextualize the results. Even a qualitative discussion of how these values relate to rodent performance would improve biological interpretability.

We agree that anchoring the reported performance metrics to experimental behaviour strengthens biological interpretability. We have therefore added qualitative discussion comparing the number of attempts required for block switches in the model to qualitative patterns observed in rodent reversal and navigation paradigms. We clarify that the goal of the present work is mechanistic understanding rather than quantitative model fitting, but note that the rapid adaptation under high contextual discriminability and slower adjustment under ambiguity qualitatively resemble behavioural patterns reported in rodents.

Specific requests:

Line 463 (line 473 in revised manuscript): We have added qualitative comparison to rodent reversal behaviour when reporting block-switch performance, clarifying how the number of attempts observed in the model relates to behavioural adaptation in T-maze paradigms.

Line 487 (line 503 in revised manuscript): We have added qualitative comparison to rodent performance when reporting both block-switch adaptation and asymptotic within-trial accuracy, clarifying how the quantitative values observed in the simulations relate to behavioural performance levels in cue-guided navigation tasks.

Line 649 (line 664 in revised manuscript): As this paragraph focuses on the internal posterior dynamics of the model rather than on reported behavioural performance metrics, we have retained the mechanistic explanation. Behavioural contextualisation of block-switch adaptation is now provided at the first reporting of attempts.

Line 722 (line 739 in revised manuscript): This section compares a training manipulation commonly used in rodent experiments (blocking access to the incorrect arm) with the corresponding manipulation in the model. As no quantitative performance metrics are introduced in this paragraph, and rodent methodology is already cited, we have retained the explanation as written, and focussed our comparisons in other parts of the manuscript.

Minor Comments

Feature representation and architectural assumptions
(Line 352; Line 430)
The scaling of cue features (e.g., factor of 4) appears important for model performance. It would be helpful to state explicitly whether this scaling is necessary for FI to function robustly and how it should be interpreted biologically.

We thank the reviewer for this clarification. We have added a sentence at Line 352 stating that the factor of 4 scales the cue feature relative to spatial state features to ensure sufficient discriminability between trial types in the baseline regime. We clarify that this parameter controls relative feature magnitude in the model and is not intended as a biologically literal value.

Relatedly, clarifying why OI is restricted from accessing cue information (architectural assumption vs biological motivation) would improve transparency.

We have added a clarifying sentence in the Methods section after line 257 explicitly stating that OI does not receive cue features by design. We clarify that this is an architectural modelling choice introduced to isolate outcome-driven contextual labelling from feature-driven predictive mapping, allowing their interaction to be analysed mechanistically. This restriction is not intended as a strict biological claim about circuit-level access to sensory information.

Specific requests:

Line 352 (line 356 in revised manuscript): Clarified as described above, specifying that the cue scaling controls discriminability in the baseline regime and is not a biologically literal parameter.

Line 430 (line 439 in revised manuscript): This section was revised in response to earlier comments regarding task framing and comparison to existing contextual inference approaches. We clarified that the cued T-maze serves as a high-discriminability baseline regime and moderated language suggesting general benchmarking against alternative algorithms.

Interpretation of results versus model design
(Line 495)
The statement that FI agents “formed distinct successor maps” appears largely driven by model design. Reframing this as a mechanism that enables separation—rather than as an empirical discovery—would improve precision.

We agree with the reviewer and have revised the wording at **Line 495 (509 in revised manuscript)** to clarify that separation of successor maps is enabled by the model architecture through context-dependent gating, rather than implying that this separation emerged as an empirical discovery. The revised text now frames map separation as a designed mechanism whose stability depends on contextual discriminability.

Successor representation flexibility
(Line 1157)
The discussion of SR flexibility appears to conflate reward revaluation with transition structure changes. Since classical SR is not flexible to changes in transition dynamics without recomputation, moderating this claim would improve technical accuracy.

We thank the reviewer for this clarification. We have revised the sentence at **Line 1157 (line 1227 in revised manuscript)** to distinguish explicitly between reward revaluation and changes in transition structure. The revised text now states that successor representations support rapid adaptation to reward revaluation, whereas changes in transition dynamics require updating of the predictive map. This corrects the error in the previous wording.

Inference dynamics and observability
(Lines 1209, 1296)
Inference in the model occurs continuously at each state, whereas biologically inference is often event-triggered (e.g., at cue onset or outcome). Acknowledging this distinction would strengthen the discussion. Similarly, the cue is fully informative but temporally displaced from action, which may be more accurately described as delayed information rather than partial observability.

We have added discussion on decision timing on **line 1209 (line 1298 in revised manuscript)** to explicitly acknowledge that continuous state-by-state updating in the model is a simplifying computational assumption, and that biological context updating may be more strongly event-triggered (e.g., at cue onset or outcome). We have also clarified that the cued T-maze involves temporally displaced but fully informative cues, and thus represents delayed access to contextual information rather than classical partial observability.

Specific requests

Line 1209 (line 1298 in revised manuscript): This point is addressed in the Discussion, where we state that continuous state-by-state inference is a simplifying abstraction and that biological implementations likely involve a mixture of discrete events and continuous processes. We have however clarified that the cued T-maze involves temporally displaced but fully informative cues, and thus represents delayed access to contextual information rather than classical partial observability.

Line 1296 (line 1392 in revised manuscript): We have revised our wording to distinguish delayed access to contextual information from classical partial observability in which information is intrinsically incomplete.

Presentation and clarity
(Lines 518, 529, 722)
Several minor presentation issues remain, including typographical corrections, clarification of terminology, and moderation of some over-general statements. The conclusion and discussion sections could likely be shortened to improve focus and avoid over-statement.

We thank the reviewer for these suggestions. We have corrected typographical issues and clarified terminology at the indicated locations, and have moderated several over-general statements where appropriate.

With respect to shortening the Discussion and Conclusion, the manuscript has been substantially revised to address comments from all reviewers, including the addition of clarifications regarding biological interpretation, model comparison, and architectural assumptions. We have aimed to balance precision with readability while avoiding overstatement. We are, of course, happy to further condense these sections at the Editors’ discretion should additional streamlining be required.
